# SAM-VI riboswitch structure and signature for ligand discrimination

**DOI:** 10.1038/s41467-019-13600-9

**Published:** 2019-12-16

**Authors:** Aiai Sun, Catherina Gasser, Fudong Li, Hao Chen, Stefan Mair, Olga Krasheninina, Ronald Micura, Aiming Ren

**Affiliations:** 1 Life Sciences Institute, Zhejiang University, 310058 Hangzhou, Zhejiang China; 2 Institute of Organic Chemistry, Center for Molecular Biosciences Innsbruck, Leopold Franzens University, Innsbruck, A6020 Austria; 3 National Science Center for Physical Sciences at Microscale Division of Molecular & Cell Biophysics and School of Life Sciences, University of Science and Technology of China, 230026 Hefei, China

**Keywords:** RNA, X-ray crystallography, Nucleic acids

## Abstract

Riboswitches are metabolite-sensing, conserved domains located in non-coding regions of mRNA that are central to regulation of gene expression. Here we report the first three-dimensional structure of the recently discovered *S*-adenosyl-L-methionine responsive SAM-VI riboswitch. SAM-VI adopts a unique fold and ligand pocket that are distinct from all other known SAM riboswitch classes. The ligand binds to the junctional region with its adenine tightly intercalated and Hoogsteen base-paired. Furthermore, we reveal the ligand discrimination mode of SAM-VI by additional X-ray structures of this riboswitch bound to *S*-adenosyl-L-homocysteine and a synthetic ligand mimic, in combination with isothermal titration calorimetry and fluorescence spectroscopy to explore binding thermodynamics and kinetics. The structure is further evaluated by analysis of ligand binding to SAM-VI mutants. It thus provides a thorough basis for developing synthetic SAM cofactors for applications in chemical and synthetic RNA biology.

## Abstract (web summary)

Riboswitches are conserved RNA domains located in the non-coding region of mRNA that recognize cellular metabolites and, in turn, regulate gene expression. Here the authors report the structure of the recently identified SAM-VI riboswitch and provide insight into its mechanism of ligand discrimination.

## Introduction

Riboswitches are gene regulatory elements commonly located in the 5’-untranslated regions (5′-UTRs) of bacterial mRNAs^1,2^. They consist of two functional domains, the ligand-sensing aptamer and the downstream adjoining expression platform. The aptamer is able to bind the cognate ligand with high affinity and specificity, which consequently induces conformational changes that are transduced into the expression platform. This, in turn, results in transcriptional or translational regulation of the expression of downstream genes^2–4^. The genes usually encode for proteins involved in the production and transport of the metabolite that itself binds to the riboswitch. Therefore, sensing and binding of its own metabolite by the riboswitch acts as a feedback to control gene expression. Since the first description of riboswitches^5–8^, more than forty riboswitches for different ligand types have been identified in nature^3,9^. Among these riboswitches, distinct riboswitch folds (commonly referred to as ‘riboswitch classes’) can recognize the same cognate ligand, such as for cyclic-di-GMP, pre-queuosine-1 base (preQ_1_), guanidine, or *S*-adenosyl-L-methionine (SAM)^3^.

SAM is an essential metabolite that serves as a co-factor in many different enzymatic reactions. It is synthesized from ATP and methionine by SAM synthetase. Characteristic for its chemical structure (shown in Supplementary Fig. 1a) is a positively charged sulfonium group carrying a methyl, an aminocarboxypropyl and an adenosyl group. Most commonly, enzymes use SAM as methyl donor and transfer the methyl group to a broad spectrum of substrates, ranging from small molecules to proteins to nucleic acids. These enzymes termed methyltransferases (MTases) are numerous, highly specialized, and encountered in all domains of life^10^. In the course of the methylation reaction, SAM is converted to *S*-adenosylhomocysteine (SAH) with a neutral thioether instead of the original sulfonium moiety (Supplementary Fig. 1a). Importantly, the intracellular SAM concentration is tightly regulated, and in bacteria, this task is frequently handled by riboswitches^11^. Likely because of the universal biological importance of SAM, the SAM riboswitches are among the most abundant riboswitches^11,12^. According to the structural, sequence and evolutionary relatedness, SAM riboswitches fall into different classes and families^3,11–14^. SAM-I/S box^15–18^, SAM-IV^19^, and SAM-I/IV^20^ classes are grouped as SAM-I family, while SAM-II^21^ and SAM-V^22^ are grouped as SAM-II family. SAM-III, originally called S_MK_ box^23^, defines the SAM-III family^14^. The SAM-I/II/III families all strongly discriminate SAM over SAH. Riboswitches that preferentially bind to SAH and discriminate against SAM are also known and constitute the SAH class^24^. Interestingly, one riboswitch class binds to SAM and SAH with similar binding affinity and therefore represents the first member of a SAH/SAM family^20^. In particular the SAM-riboswitch families provide a perfect setting for investigations on how the same cognate ligand can be recognized by different RNA architectures. Towards this end, X-ray crystallographic and NMR-spectroscopic structural research has made significant contributions^25–30^ to shed light on the distinct ligand recognition modes, which provide the basis for the cellular function of SAM riboswitches.

Recently, a new SAM-riboswitch class, termed SAM-VI, has been identified in species of *Bifidobacterium* by Breaker and co-workers using computational methods of comparative sequence analysis^13^. The conserved sequence and secondary structure of SAM-VI (Supplementary Fig. 1b) has vague similarities with the SAM-III riboswitch. Both SAM-III and SAM-VI consist of three stems (P1, P2, and P3) that are connected by one central 3-way junction, and both riboswitches also selectively bind SAM over SAH. Furthermore, the nucleotides that interact with the ligand in SAM-III are also present in SAM-VI (Supplementary Fig. 1c) and the Shine-Dalgarno (SD) sequence joins stems P1 and P3 in both secondary structure models. However, SAM-VI has a different phylogenetic distribution compared to SAM-III. Distinct from SAM-III is that the SAM-VI secondary structure model integrates the AUG start site in the terminal part of stem P1 (Supplementary Fig. 1b). The SAM-VI model displays no bulge in stem P2, which is known to be crucial for the formation of the SAM-III binding pocket. Besides, SAM-VI has six compared to three nucleotides in junction J2-3, with all of them being highly conserved. In addition, many more nucleotides are uniquely conserved in the consensus sequence of SAM-VI compared to SAM-III^13^.

To reveal the architecture of the SAM-VI riboswitch and to illustrate the selectivity and the recognition mode of ligand, we set out to solve its three-dimensional structure using X-ray crystallography. Here, we describe the 2.7 Å resolution crystal structure of SAM-VI bound to its cognate ligand SAM, complemented by structures of SAM-VI bound to SAH (3.1 Å resolution) and one synthetic ligand analog (2.8 Å resolution). Furthermore, we used mutational analysis, isothermal titration calorimetry (ITC) and fluorescence spectroscopy for a critical evaluation of the novel RNA fold and to obtain selected thermodynamics and kinetics parameters for ligand binding. This comprehensive information allows us to present a model for the action of this RNA as a translational OFF switch. Our study thus paves the way for future developments towards potential applications in chemical and synthetic biology, as well as in biotechnology and biomedicine. In particular, we believe that the thorough structural basis we provide here can be key for the design of RNA labeling tools. To this end, in vitro selection approaches that would utilize SAM-riboswitch scaffolds in combination with synthetic co-factor analogs (e.g., *S*-propargyl SAM derivatives) may deliver protein-free tools for covalent labeling of cellular RNA targets^31^.

## Results

### Design of SAM-VI RNA constructs for structure determination

**Fig. 1:**
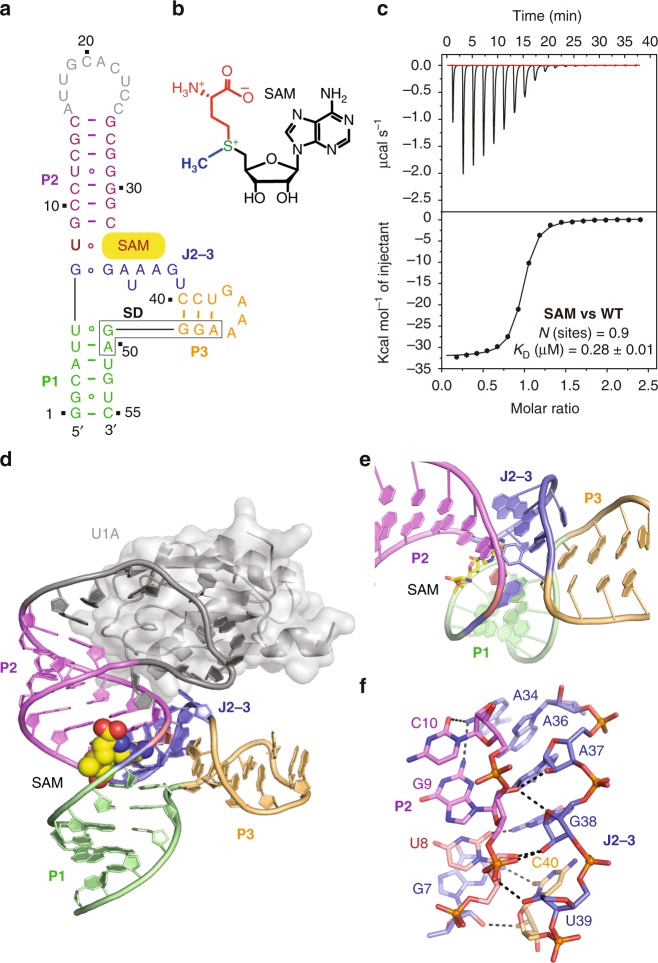
Secondary and tertiary structure of the *B. angulatum* SAM-VI riboswitch. **a** Schematic of the folding topology based on the crystal structure of the SAM-VI riboswitch; the Shine-Dalgarno sequence is highlighted (box). **b** Chemical structure of SAM. **c** Exemplary ITC titration experiment of SAM-VI riboswitch binding with SAM; for arithmetic mean of *K*_d_ value and thermodynamic parameters see Table 1 and Supplementary Table 2. **d** Cartoon representation of 2.7 Å structure of the SAM-VI riboswitch, color-coded as in **a**. **e** Expanded view on the SAM-VI junction harboring the binding pocket. Stems P1 and P2 are co-axially stacked under the assistance of SAM adenine and junctional nucleotides involved in base pairing at the P1-P2 interface. P3 is located perpendicular to the P1-P2 axis and not directly involved in pocket formation. **f** Ribose and backbone interactions between stem P2 and junction J2-3 (of four consecutive nucleotides each) shape the pocket.

The secondary structure model for the SAM-VI riboswitch predicts the formation of three stems P1, P2, and P3 that are connected by one central 3-way junction and that do not contribute to formation of any obvious long-range interactions (Supplementary Fig. 1b)^13^. We screened a large number of in vitro transcribed SAM-VI riboswitch sequences and constructs, in which we changed the sequence of the variable loop and the length of stems P1 and P2 to facilitate crystallization. One transcript from *B. angulatum* 59 *metK* in which the U1A recognition site had been introduced as terminal loop of stem P2 (and the non-conserved G42 had been mutated to U42) yielded diffraction quality crystals when co-crystallized with the U1A protein (Fig. 1a). We solved the structure with single-wavelength anomalous diffraction (SAD) phasing by collecting the anomalous signal of selenium in selenomethionine (SeMet)-derivatized U1A protein used in co-crystallization (Supplementary Table 1). The electron-density calculated from phases of the final refined model of SAM-VI riboswitch is shown in Supplementary Fig. 2a (for clarity, the electron-density of the U1A protein and the RNA binding site was omitted). Isothermal titration calorimetry (ITC) experiments revealed that the RNA sequence we used to solve the structure binds SAM (chemical structure shown in Fig. 1b) with an affinity constant *K*_d_ of 0.33 µM (Δ*G* = −8.7 kcal/mol) (Fig. 1c, Table 1, and Supplementary Table 2).

**Table 1:** Thermodynamic and kinetic parameters of ligand binding to SAM-VI^a^.

	K_d_ (ITC) 293 K (µM)^b^	k_on_ 293 K (M^−1^s^−1^)
SAM-VI		
SAM	0.33 ± 0.06	24,580 ± 689
SAH	10.9 ± 1.3	5500 ± 266
Ligand M1	4.4 ± 0.5	17,240 ± 1140
U6C SAM-VI		
SAM	0.46 ± 0.02	62,550 ± 1900
SAH	2.3 ± 0.1	1460 ± 179
Ligand M1	1.7 ± 0.3	4230 ± 462

^a^For an extension of Table 1 containing data of individual replicates, and a comparison to other SAM-riboswitch classes, see the Supplementary Information (Supplementary Tables 2 and 3)

^b^Values are the arithmetic mean of three independent experiments; errors are standard deviation

### Tertiary fold of SAM-VI RNA bound to SAM

The schematic second structure and the underlying tertiary structure of the SAM-bound SAM-VI riboswitch are shown in Fig. 1a, d. The SAM-VI fold is composed of three stems P1, P2, and P3 that are consistent with the originally predicted secondary structure (Supplementary Fig. 1b). Stem P2 (in violet) stacks co-axially with stem P1 (in green), mediated by two intercalating base pairs from the junctional region of the riboswitch and the ligand SAM (Supplementary Fig. 2b). One is the non-canonical base pair formed by the residues G7 and G33 (from J1-2 and J2-3). The other base pair was formed by U8 and the SAM-adenine base (Fig. 1a). Stem P3 is positioned almost perpendicular to the long helix axis formed by stems P1 and P2 (Fig. 1d, e). Importantly, junction J2-3 (G33-U39) folds very close to one of the chains of the P2 double helix. Three out of the seven nucleotides in this junction (A37-U39) and the following C40 exhibit extensive hydrogen-bonding interactions with the stem P2 nucleotides G7-C10, thereby dominantly involving their ribose 2′-OH groups (Fig. 1f and Supplementary Fig. 3). This motif represents a variation of the ribose zipper^32,33^ with the 2′-OHs of G7-U8-G9 directly interacting with the 2′-OHs of A37-G38 and C40, complemented by one interaction in between, namely an H-bond of the G9 phosphate and the 2′-OH of U39 (Fig. 1f). The SAM-binding site of the SAM-VI riboswitch is located in the 3-way junctional region between stems P1 and P2, and does not border upon stem P3 (Fig. 1a, d, e). Notably, the ligand integrates itself from the major groove side of P1 and P2 (Fig. 1d, e).

### Nucleoside alignments in the binding pocket

**Fig. 2:**
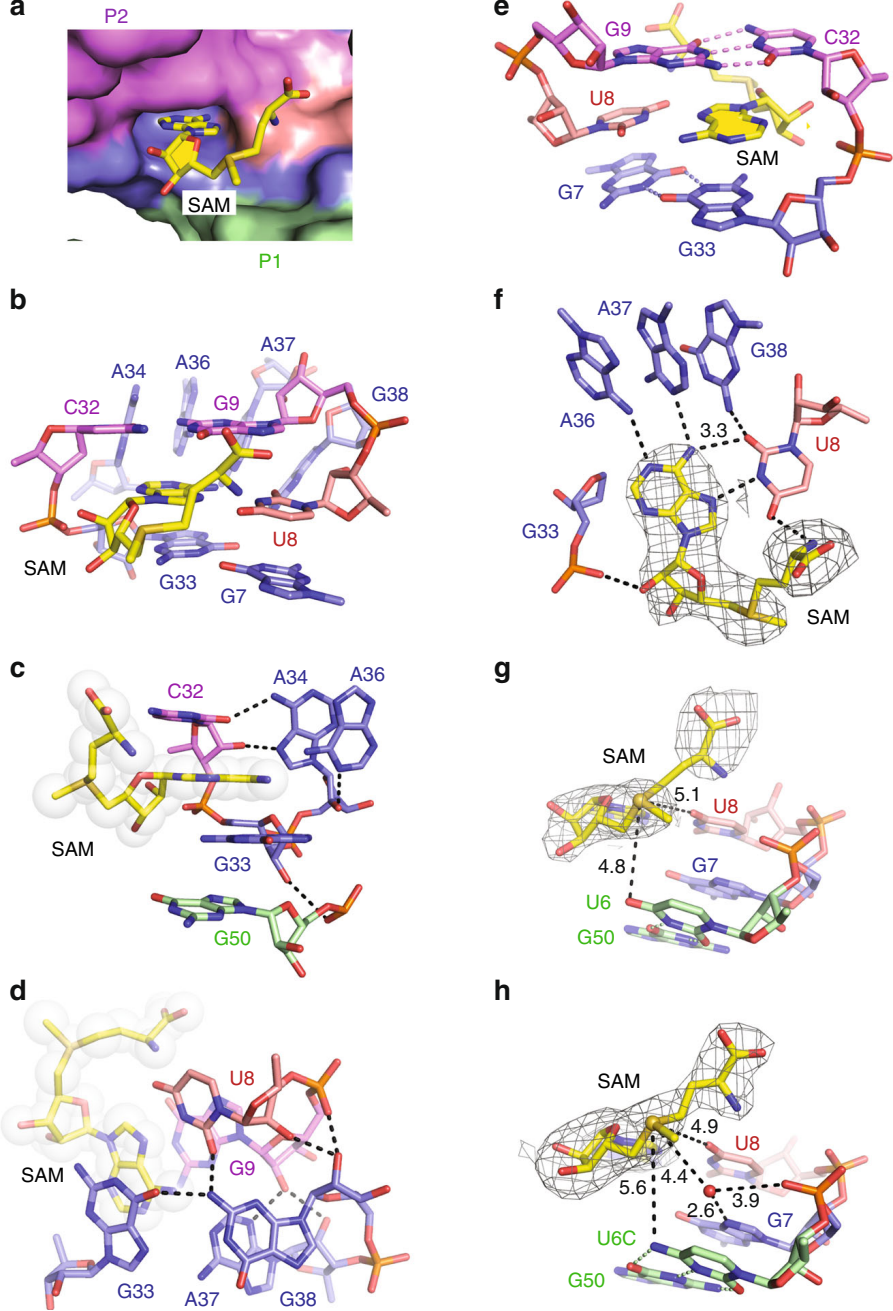
Structural details of the SAM-VI riboswitch pocket bound to SAM. **a** RNA surface representation shows the cavity for the adenine base of the SAM ligand buried into the RNA and the methionine moiety directed outwards. **b**–**e** Different views on the binding pocket interactions with ligand SAM (for discussion see main text). **f** Detailed view of the SAM-adenine U8 base pair. The composite omit electron-density map of SAM contoured at the 1.0 *σ* level is shown in light gray. **g** Detailed view on the sulfonium moiety and the nearest neighbors (O4 U6 and O4 U8) with selected distances indicated by dotted lines and corresponding values in Å. The composite omit electron-density map of SAM contoured at the 1.0 *σ* level is shown in light grey. **h** Binding pocket of the U6C-mutant SAM-VI riboswitch: U6C moves away from the sulfonium moiety; note that a water molecule appears and takes over the role of the mutated U6; distance values are in Å. The composite omit electron-density map of SAM contoured at the 1.0 *σ* level is shown in light gray.

The surface representation of the SAM-VI pocket (Fig. 2a) shows that the adenine base of SAM (shown in sticks) is intercalating and becomes stacked, while the sulfonium moiety and the methionine tail point outward from the adenine intercalation site. The binding pocket itself is composed of one terminal Watson-Crick base pair G9-C32 from stem P2 (Fig. 2b), one non-canonical (*trans* Watson-Crick) base pair G7•G33 (Fig. 2b) from J1-2 and J2-3, four continuously stacked residues A34, A36, A37, and G38 from J2-3 (Fig. 2b), and importantly, the U8 (from J1-2) that recognizes the SAM-adenine base (Fig. 2b).

Further detailed inspection of the binding pocket reveals that SAM is bracketed tightly by three consecutive residues C32-G33-A34 from three sides along the Watson-Crick edge of its adenine base (Fig. 2c). C32 and G33 sandwich the SAM-adenine base and stack on it from both sides. The base and sugar of C32 additionally form two hydrogen bonds with the Hoogsteen edge of A34. A34 itself stacks with A36. At the same time, the 2′-OH of A34 hydrogen–bonds with N1 of A36 and the 2′-OH of G33 forms one hydrogen bond with the non-bridging phosphate-oxygen of the G33-stacked G50 from stem P1. Notably, G33 and A34 both adopt *C2*′*-endo* ribose pucker conformation.

The SAM ligand is further locked by two other sections of consecutive residues, U8-G9 and A37-G38, which interact with each other (Fig. 2d): the motif is stabilized by A37 forming hydrogen bonds with G9 (A37 N3 and 2′-OH with the G9 2′-OH), and by a further one between the non-bridging phosphate-oxygen of G9 and the 2′-OH of G38. Furthermore, the base of G38 forms a hydrogen bond with U8 (2-NH_2_ G38 with O2 U8), and an additional one to the base of G33 (2-NH_2_ G38 with O6 G33).

The SAM ligand adopts a C3′-*endo* ribose conformation and the SAM-adenine base is in *anti* conformation at the glycosidic bond when bound to SAM-VI RNA. Thereby, SAM interacts intensively with the binding pocket through stacking (Fig. 2b, e) and hydrogen-bonding (Fig. 2f). The terminal base pair G9-C32 of stem P2 and the *trans* Watson–Crick pair G7•G33 from the central junction stack on the two sides of the SAM-adenine base (Fig. 2e). The Hoogsteen edge of the SAM-adenine pairs with the Watson-Crick edge of U8 (Fig. 2f). Furthermore, 6-NH_2_ of A36 and N1 of A37 also form one hydrogen bond each with the Watson-Crick edge of the adenine base of SAM (Fig. 2f). We note that A36, A37, and G38 stack continuously on each other. G38 does not form direct interactions with SAM, but forms one hydrogen bond with O2 of U8 that pairs with the SAM-adenine base, which may augment the interaction between SAM and the binding pocket of SAM-VI riboswitch. The non-bridging phosphate-oxygen of G33 forms one hydrogen bond with the 2′-OH of the ribose sugar of SAM (Fig. 2f). Notably, the methionine tail is structured and folds back, supported by an H-bond interaction between the α-amino group of the SAM methionine and O4 of U8 (Fig. 2f). The composite omit map of ligand SAM is shown in Fig. 2f.

We further note that the sulfonium moiety of SAM is in the vicinity of the O4 atom of the U6 nucleobase (4.8 Å, Fig. 2g). This pyrimidine forms a wobble base pair with G50 (Fig. 2g) at the terminus of P1 towards the central junction, which is highly conserved in the SAM-VI consensus sequence (Supplementary Fig. 1b, c). The O4 of U6 likely assists in recognition and stabilization of the positive charge of the sulfonium moiety. At this point, we note that spatial orientation of oxygen atoms toward the sulfonium moiety is also observed for other SAM-riboswitch classes. In SAM-I, O4s of two partially stacked uridines, U7 and U88, point at the sulfonium with distances of 4.3 and 4.0 Å (Supplementary Fig. 4a)^26,34^. Similarly, for the SAM-III family, one uridine O4 and one ribose 2′-O approach the sulfonium in the 4 Å range (Supplementary Fig. 4c)^27^. This recognition strategy is even more pronounced for SAM-II and SAM-V, where two uridines orientate their O4 carbonyl oxygens from opposite sides in only 3.2 and 3.3 Å distances toward the sulfonium (Supplementary Fig. 4b, d)^26,28^.

### Mutational and calorimetric analysis

As discussed above, the conserved nucleotides in the SAM-VI RNA sequence contribute significantly to stabilization of the global riboswitch fold and the recognition of its cognate ligand. To evaluate the crystal structure, we performed mutational analysis in combination with isothermal titration calorimetry (ITC) to assign the impact of individual key residues. Using the same RNA construct as for crystallization, we found that wild-type SAM-VI binds SAM with low micromolar affinity under our experimental conditions (*K*_d_ of 0.33 µM, Δ*G* = −8.7 kcal/mol; Supplementary Table 2 and Supplementary Fig. 5a). Mutation of U8 (that forms vital interaction with the SAM-adenine base and interacts with the α-amino group of the SAM methionine moiety and with G38) to C, A, and G respectively, resulted in a loss of binding for all three riboswitch mutants (Supplementary Table 2 and Supplementary Fig. 6a). G33 stacks with the adenine base of SAM and pairs in *trans* Watson–Crick mode with G7, thereby forming the floor of the binding pocket (Fig. 2e). To assess the impact of G33 on ligand binding, we mutated G33 to A33. Not unexpectedly, the G33A mutant did no longer bind to SAM (Supplementary Table 2 and Supplementary Fig. 6b). The base of A34 stacks with A36 and hydrogen-bonds with C32, which brackets one end of the SAM ligand (Fig. 2c). To test the importance of A34 for this structural arrangement, we investigated two mutations, namely A34G and A34C. For both the A34G and A34C mutants, binding affinities were below detection (Supplementary Table 2 and Supplementary Fig. 6b). This is consistent with the loss of the specific hydrogen-bonding interaction between A34 and C32 and with the reduced stacking capacity of pyrimidines compared to purines (Fig. 2c). The three consecutive residues A36, A37, and G38 stack continuously and form hydrogen bonds with the Watson–Crick edge of the adenine base of SAM and the O2 of U8 in the ligand-binding pocket (Fig. 2f). We individually mutated these residues to C and all three riboswitch mutants displayed no binding activity (Supplementary Table 2 and Supplementary Fig. 6b). In addition, it was found that the SAM-VI variant with triple mutation A36G/A37G/G38A in order to retain stacking but to change the hydrogen acceptor-donor pattern did hinder binding similarly (Supplementary Table 2 and Supplementary Fig. 6b). Taken together, the combined mutational and calorimetric analysis validates the obtained crystal structure of the SAM-VI riboswitch.

### Structure of SAM-VI RNA bound to SAH

**Fig. 3:**
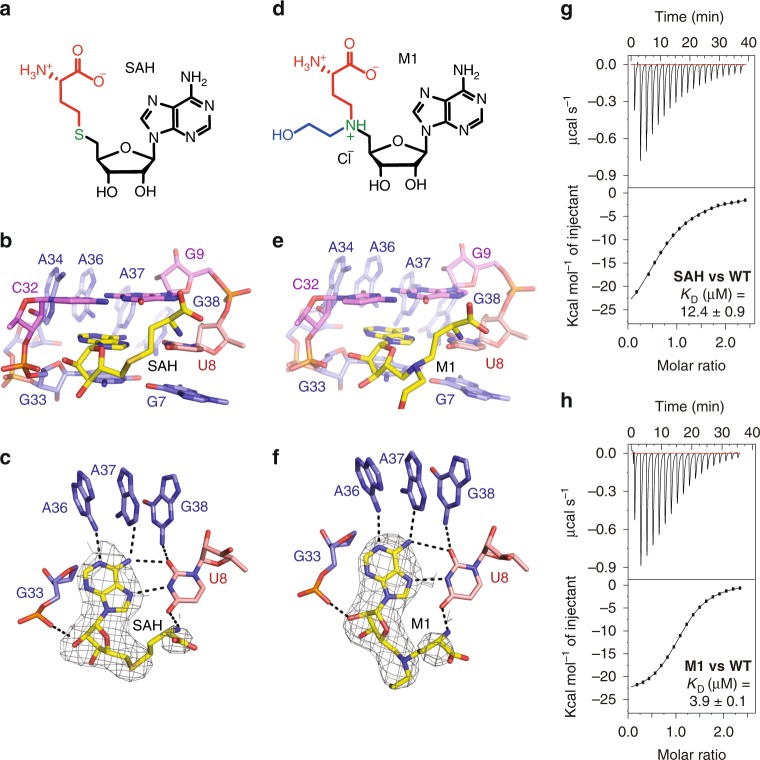
Structures of SAM-VI riboswitch bound to non-cognate SAH and a ligand mimic. **a** Chemical structure of SAH. **b** SAM-VI binding pocket interactions with SAH (for discussion see main text). **c** Detailed view of the SAH-adenine U8 base pair. The composite omit electron-density map of SAH contoured at the 1.0 *σ* level is shown in light gray. **d** Chemical structure of the ligand mimic (M1). **e** SAM-VI binding pocket interactions with M1 (for discussion see main text). **f** Detailed view of the M1-adenine U8 base pair. The composite omit electron-density map of M1 contoured at the 1.0 *σ* level is shown in light gray. **g**, **h** Exemplary ITC titration experiments of SAM-VI riboswitch binding with SAH and the ligand mimic M1, respectively; for arithmetic means of *K*_d_ values and thermodynamic parameters see Table 1 and Supplementary Table 2.

The SAM-VI riboswitch can also bind to the demethylation product of SAM, that is SAH, although the riboswitch affinity to SAH is reduced in comparison to the cognate ligand^13^. Isothermal titration calorimetry (ITC) gave a roughly 33-fold decreased dissociation constant *K*_d_ of 10.9 μM for SAH compared to SAM (*K*_d_ of 0.33 µM) (Supplementary Table 2 and Fig. 3a, b, g), which is equivalent with the binding free energy difference (ΔΔ*G* = 2.0 kcal/mol) between SAH (Δ*G* = −6.7 kcal/mol) and SAM (Δ*G* = −8.7 kcal/mol) to wild-type SAM-VI riboswitch. The discrimination of SAM over SAH binding is therefore consistent with the original report in the literature^13^. Notably, the discrimination is in the same order as encountered for SAM-I RNA (80-fold)^15,35^ and SAM-III RNA (100-fold)^27,35,36^ while SAM-II RNA discriminates SAM more than 1000-fold over SAH^35,37^ (Supplementary Table 3).

Encouraged by these results, we attempted co-crystallization of SAM-VI RNA with SAH and we obtained well-diffracting crystals under similar crystallization conditions. The SAH-bound riboswitch structure was solved using molecular replacement (MR) with the SAM-VI/SAM complex as the structural model.

The SAM-binding pocket of SAM-VI riboswitch retains its architecture also when bound to SAH (Fig. 3b, c). The adenine base of SAH is stacked between G9-C32 and G33•G7 as observed for SAM binding (Fig. 2e). Likewise, A34, A36, A37, and G38 still form a stacked base column and bracket the nucleobase side of SAH (Fig. 3b, c). The adenine base itself pairs with U8 via the Hoogsteen edge and with A36 and A37 via the Watson-Crick edge (Fig. 3c). The homocysteine moiety of SAH points outward from the binding pocket (Fig. 3b), but folds back with its amino group forming a hydrogen bond with O4 of U8 (Fig. 3c). All these interactions of the binding pocket with SAH are similar to the SAM-binding mode and the question arises what could be the structural reason for the discrimination of SAM over SAH in the SAM-VI motif and for the difference in binding affinities. As shown in Fig. 2g, the closest residues to the positively charged sulfonium moiety are U8 and U6 with O4 of U8 and O4 of U6 located in 5.1 and 4.8 Å distance, respectively. As mentioned above, when U8 was mutated to C, SAM-VI lost binding capability; however, this effect is mostly attributed to the loss of proper placement of the SAM-adenine moiety in the binding pocket (Fig. 2e, f and Supplementary Fig. 6b). To explore the potential impact of U6, we investigated a U6C riboswitch mutant. This mutation alters the wild-type U6•G50 wobble pair into a standard C–G base pair in P1 (Fig. 2h). Interestingly, this mutant binds SAM with almost similar affinity (*K*_d_ of 0.46 µM, Δ*G* = −8.5 kcal/mol, ΔΔ*G* = 0.2 kcal/mol, Supplementary Table 2 and Supplementary Fig. 7a) as the wild-type RNA (*K*_d_ of 0.33 µM, Fig. 1c); however, its discrimination over SAH was only 5-fold (*K*_d_ of 2.3 µM, Δ*G* = −7.6 kcal/mol, Supplementary Table 2 and Supplementary Fig. 7b) compared to 33-fold in the case of wild-type RNA (Supplementary Table 2).

To further explore the recognition mode of the sulfonium moiety, we also solved the crystal structure of the U6C-mutant RNA bound to SAM. The overall fold, the binding pocket composition, and the SAM-adenine pairing to U8 are comparable to the wild-type complex (Supplementary Fig. 8). Superposition of the wild-type and U6C-mutant complex structures in PyMOL^38^ generated a root mean square deviation (rmsd) of 0.43 Å (Supplementary Fig. 8a). Relative to the O4 atom of U8, the sulfonium moiety is hardly shifted, with a distance decreasing from 5.1 Å to 4.9 Å (being within the coordinate error) (Fig. 2g, h and Supplementary Fig. 8b). At the same time, C6 (originally U6) moves away from the sulfonium, with O4 versus 4-NH_2_ to S^+^ distances significantly shifting from 4.8 Å to 5.6 Å (Fig. 2g, h and Supplementary Fig. 8b). Strikingly, the lack of the O4-U6 interaction became compensated by one water molecule that is hydrogen-bonded to the N7 of G7 (2.6 Å) and directed toward the sulfonium moiety (4.4 Å) in the U6C complex (Fig. 2h and Supplementary Fig. 8b). The composite omit electron-density map (contoured at 1.0 σ level) of SAM and the involved residues U6/C6, G7, U8, and G50 are shown in Fig. 2g, h and Supplementary Fig. 8c, d. These observations also shed light on the molecular determinants for discrimination of SAM versus SAH: While the electrostatic interactions between oxygen atoms and the sulfonium moiety are thermodynamically favourable and contribute to SAM binding, the thioether moiety of SAH results in repulsive lone pair interactions with the oxygens O4-U6 and O4-U8 in wild-type SAM-VI to discriminate SAH against SAM. These considerations are consistent with the obtained ITC *K*_d_ values and the corresponding Gibbs free energy changes (Supplementary Table 2) and the high conservation of the U6•G50 wobble base pair (Supplementary Fig. 1b).

### Structure of SAM-VI RNA bound to a N-mustard SAM analog

SAM/SAH analogs have received considerable interest as versatile tools in combination with SAM-dependent methyltransferases (MTases)^39^. The major function of SAM-dependent MTases is to transfer the methyl group of the co-factor SAM to a target biomolecule. The resultant transmethylation constitutes a fundamental molecular mechanism in cells, providing the basis for epigenetic regulation, cellular signaling, and metabolite degradation. Numerous SAM analogs have been reported as synthetic cofactors to transfer the activated groups on MTase substrates for downstream ligation and identification. Additionally, SAM/SAH analogs have been designed and tested as selective inhibitors for important MTase targets. However, only little is known about SAM/SAH analogs as inhibitors or as synthetic biology tools in conjunction with SAM-sensing RNA riboswitches. Only few reports provide information about SAM-riboswitch binding to analogs that use the co-factor scaffold^35,40,41^ and to the best of our knowledge, none is available that utilizes the potential alkyl transfer reactivity of SAM analogs in riboswitch-engineered systems. Studies towards that aim would not only impact the field of synthetic RNA biology but also tremendously impact the RNA world hypothesis^42^.

To generally contribute to the above mentioned aspects, and in particular, to shed further light on the SAM-VI ligand recognition mode, we considered SAM/SAH analogs comprising a tertiary amino group instead of the native sulfonium. At physiological pH and in the microenvironment of the binding pocket, the amino functionality might become protonated and such analogs could therefore constitute ligands competitive to SAM, while at higher pH values their behavior likely resembles that of neutral SAH. We therefore synthesized *N*-mustard analogs of SAM in analogy to reports in the literature^43–45^ (Supplementary Figs 9–13). Eventually, we were able to crystallize the SAM-VI construct with one such compound (M1) whose chemical structure is shown in Fig. 3d. In M1, besides the S-to-N exchange, the original methyl group is replaced by a *N*-(2-hydroxyethyl) group (Fig. 3d). This compound binds 13-fold weaker compared to SAM, with a *K*_d_ of 4.4 µM (Supplementary Table 2 and Supplementary Fig. 5c). A close-up of the M1 occupied pocket bound of SAM-VI is shown in Fig. 3e, f. Ligand M1 stacks and hydrogen bonds in comparable manner as observed for SAM or SAH and the majority of intermolecular contacts are retained (Fig. 3f). Under the crystallization conditions used (pH value of 4.7), protonation of the amine is likely. No direct H-bond interactions of the ammonium moiety with the RNA are observed and this suggests an electrostatic stabilization comparable to the sulfonium moiety of the native ligand.

### Kinetics of ligand binding to SAM-VI RNA

**Fig. 4:**
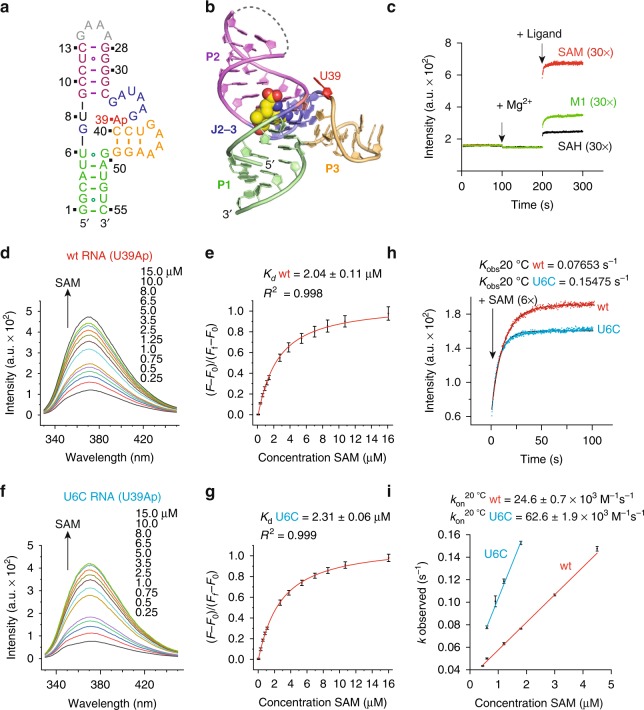
Kinetics of ligand binding to SAM-VI RNA. **a** Sequence and secondary structure of the 2-aminopurine (Ap) modified RNA used for fluorescence spectroscopic experiments. **b** The nucleoside U39 (in red) that was selected for Ap replacement is located close to the binding pocket and directed outwards. **c** Real time fluorescence traces for SAM-VI riboswitch complex formation, upon Mg^2+^ and ligand additions. **d** Fluorescence changes upon titration of Ap labeled SAM-VI riboswitch variant (wt U39Ap) with ligand SAM; fluorescence emission spectra (*λ*_ex_ = 308 nm) from 330 to 450 nm of the wt U39Ap variant for each ligand concentration. **e** Normalized fluorescence intensity of the wt U39Ap variant plotted as a function of SAM ligand concentration. The graph shows the best fit to a single­site binding model (see Methods). Changes in fluorescence (*F*-*F*_0_) were normalized to the maximum fluorescence measured in saturating concentrations of the SAM ligand. The obtained *K*_d_ (Ap) values (50 mM KMOPS, pH 7.5, 100 mM KCl, 2 mM MgCl_2_, 293 K) were slightly higher compared to the *K*_d_ values obtained from ITC titration experiments that were performed at 10 mM Mg^2+^ concentrations. **f**, **g** Same as **d**, **e** but for titration of the U6C U39Ap variant with SAM ligand. For further details, see Methods. **h** Stopped-flow fluorescence spectroscopy was used to monitor the kinetics of SAM-VI riboswitch complex formation using wild-type (wt) and U6C SAM-VI RNAs. Exemplary fluorescence traces are depicted; conditions: 0.3 μM RNA, 100 mM KCl, 50 mM MOPS, pH 7.5, 293 K. Ligands: 2 mM MgCl_2_, 1.8 μM ligand. **i** The mean *k*_obs_ values determined from three independent experiments with the corresponding error bars are plotted against the concentration of SAM and subjected to a linear fit. The slope of the plot yields the rate constant *k*_on_.

To evaluate binding thermodynamics and kinetics, we developed a 2-aminopurine (Ap) based fluorescence assay for the SAM-VI riboswitch using an approach (2ApFold) that we introduced earlier^46,47^. Based on the crystal structure of the SAM-bound RNA, we selected nucleoside position U39 for the single Ap substitution (Fig. 4a, b). U39 is close to the binding pocket and directed outwards (Fig. 4b). It was therefore expected that ligand binding can be followed in real time by monitoring the fluorescence increase originating from the nucleobase that becomes unstacked during the ligand-induced folding process. These structure-based selection criteria have proven reliable with respect to minimal interference of the nucleobase substitution on RNA–ligand binding, RNA folding, and RNA folding kinetics^37,48,49^. Distinct to the construct used for crystallization and ITC measurements, we synthesized a SAM-VI construct with stem P2 closed by a GAAA tetra-loop (instead of the U1A recognition loop) and containing the U39Ap modification (Fig. 4a). The qualitative fluorescence response of the U39Ap riboswitch variant (0.5 µM) upon addition of physiological concentrations of Mg^2+^ (2 mM), and subsequently, of the ligands SAM (15 μM), SAH (15 µM), and L1 (15 μM), respectively, are depicted in Fig. 4c. Mg^2+^ addition alone did not result in a fluorescence change, however, addition of SAM in 30-fold excess over RNA caused a pronounced fluorescence increase consistent with the conformational change of the reporter into a protruding and unstacked position. Also for the SAM analog M1 (in 30-fold excess) and for SAH (in 30-fold excess) binding was trackable in real time by the corresponding fluorescence signal. We determined the affinity of SAM to wild-type SAM-VI RNA with this alternative fluorescence spectroscopic approach (for reasons of comparison to ITC), and obtained a well comparable value in the low µM region (Fig. 4d, e; the 7–fold differentiation in *K*_d_ corresponds to a ΔΔ*G* of about ~1 kcal (1-2 hydrogen bonds)). The main application for the 2Ap SAM-VI variants, however, was the determination of binding kinetics of the three ligands. The on-rates (*k*_on_) were calculated from concentration-dependent datasets obtained from measurements using a stopped-flow apparatus (see Fig. 4h, i, Table 1, Supplementary Figs. 14–20 and Supplementary Table 3). SAM binds to wild-type SAM-VI RNA about 5 times faster compared to SAH, while the analog M1 provides an only 1.5-fold decreased on-rate. Interestingly, SAM binding to the U6C SAM-VI-mutant proceeds 2.5 times faster compared to wild-type RNA, while both SAH and M1 binding is about four times slower compared to wild-type RNA. The slower on-rate of SAM to the wild-type may originate from the geometry with the carbonyl O4 of the U6•G50 pair protruding farther into the ligand entry path compared to N4 of C6-G50 in the mutant.

For the ligand analog M1, we additionally tested if the pH value of the buffer solution has an impact on binding kinetics. Decreasing the pH from 7.5 to 6.0 increased the on-rate from 17240 M^−1^s^−1^ to 25940 M^−1^s^−1^. This can be rationalized with a higher degree of protonation of the tertiary amine, and hence, an improved electrostatic interaction with the sulfonium recognition site of the pocket during the binding process.

Only for two other SAM-riboswitch classes, kinetics of ligand binding have been reported. Compared to SAM-VI, *k*_on_ is 2.7–fold faster for SAM-III^36^ while it is 2.6–fold slower for SAM-II^37^ (Supplementary Table 3).

### Model for the regulation mechanism of the SAM-VI riboswitch

**Fig. 5:**
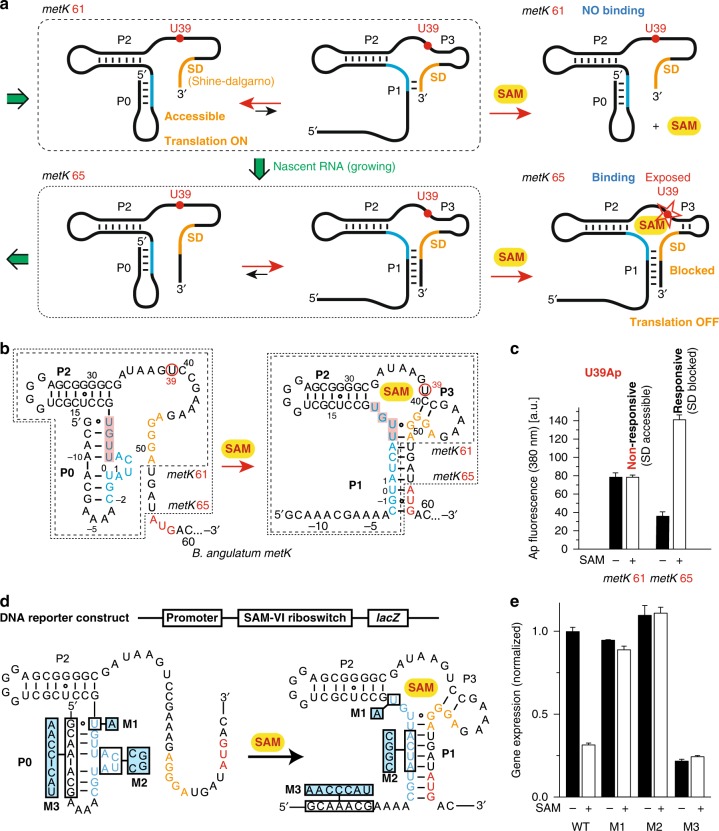
Model and experimental evaluation of SAM-VI riboswitch sequential folding and translational control. **a** Sequential folding path highlighted for two transcriptional intermediates of critical length, *metK* 61 and *metK* 65; mutually exclusive secondary structures with competing P0 and P1 stems are shown and the expected response in the presence of SAM. **b** RNA sequences used for the 2-aminopurine fluorescence assays. **c** Fluorescence changes of UAp39 labeled SAM-VI riboswitch variants (*metK* 61 and *metK* 65; c(RNA) = 0.5 µM) upon addition of saturating concentration of ligand SAM (15 µM). **d** Organization of SAM-VI riboswitch *lacZ* reporter construct in *E. coli* and RNA sequences with mutations indicated used for β-galactosidase assays. **e** In vivo expression analysis of the wild-type and mutant *E. coli metK* RNAs. ß-Galactosidase activities are presented as normalized gene expression relative to WT. Representative results of three experiments are shown (mean ± standard error of the mean).

We analyzed the sequence context of SAM-VI in *Bifidobacterium angulatum* and found that the leader sequence implies a sequential folding path that involves the alternative formation of a stem structure (P0) at the very 5′-end (Fig. 5 and Supplementary Fig. 21a). The proposed sequential folding path (Fig. 5a) offers a plausible explanation as to how the SD sequence remains accessible during the coupled process of transcription and translation when only low concentrations of SAM are available. At high concentrations of SAM, however, the ligand is captured by the growing nascent mRNA and stabilizes refolding of P0 into P1. Consequently, the SD sequence becomes sequestered, prohibiting ribosomal recognition and hence, turning off translation. Besides *B*. *angulatum*, we also analyzed other identified sequences of the SAM-VI motif. As shown in Supplementary Fig. 21b, they all carry potential for stem P0 formation. Although the sequence and the length of these stems differ between different species, they all engage the junctional J1-2 region in pairing (shown with red shadow in Supplementary Fig. 21), hence leaving the SD sequence well accessible for ribosomal recognition.

To experimentally support this hypothesis we tested the responsiveness of transcriptional intermediates that comprise the complete 5′ leader sequence. We first synthesized a U39Ap–labeled 61 nt long RNA (*metK* 61; Fig. 5a, b) that contains the entire SD sequence and at the same time provides all structural prerequisites to bind SAM and to sequester the SD site (Fig. 5a, b). This RNA equilibrates between two secondary structures, one forming stem P0 while the alternative comprises a short stem P1 (Fig. 5a). The *metK* 61 RNA, however, was not captured in the P1 comprising fold, according to the unchanged Ap fluorescence signal even at high concentrations of SAM (Fig. 5c and Supplementary Fig. 22). This observation is consistent with the notion that P0 formation supports accessibility of the SD sequence for ribosomal recognition. When the transcript becomes further elongated, as represented by the U39Ap–labeled 65 nt RNA *metK* 65, the P1 comprising fold was efficiently captured by SAM according to a more than three-fold increase in fluorescence (Fig. 5c).

Further evidence for a significant role of stem P0 in gene regulation originates from a cellular assay. We fused the *B. angulatum metK* SAM-VI riboswitch motif (WT) and selected mutants (M1, M2, M3) to a *lacZ* β-galactosidase reporter gene and monitored its production in response to SAM in vivo in *E. coli* (Fig. 5d). Expression of the wild-type *metK*–*lacZ* fusion was repressed about 4-fold when cells were grown in LB medium supplemented with SAM (Fig. 5e and Supplementary Fig. 22e). Serving as a control, the U8A mutant M1 displayed comparable expression levels to the wild-type, but was not responsive to SAM (Fig. 5e and Supplementary Fig. 22e). This is consistent with disruption of the Hoogsteen pair between U8 and SAM-adenine (Fig. 2e, f), and consequently, loss of SAM binding. Furthermore, M2 contained stem P0 with a mutated bulge (ACUA to CGGC) that was designed to interfere with P1 formation but not to affect P0 formation, hence leaving the SD sequence accessible. Additionally, we designed mutant M3, which, in contrast, interferes with P0 formation but should not affect P1 formation. As expected, M2 indeed exhibited high expression during growth while M3 exhibited only low expression, and both M2 and M3 failed to regulate the gene expression upon SAM addition (Fig. 5e and Supplementary Fig. 22e). Taken together, these results suggest that stem P0 plays a critical role in translational control of the SAM-VI riboswitch to regulate downstream gene expression.

## Discussion

**Fig. 6:**
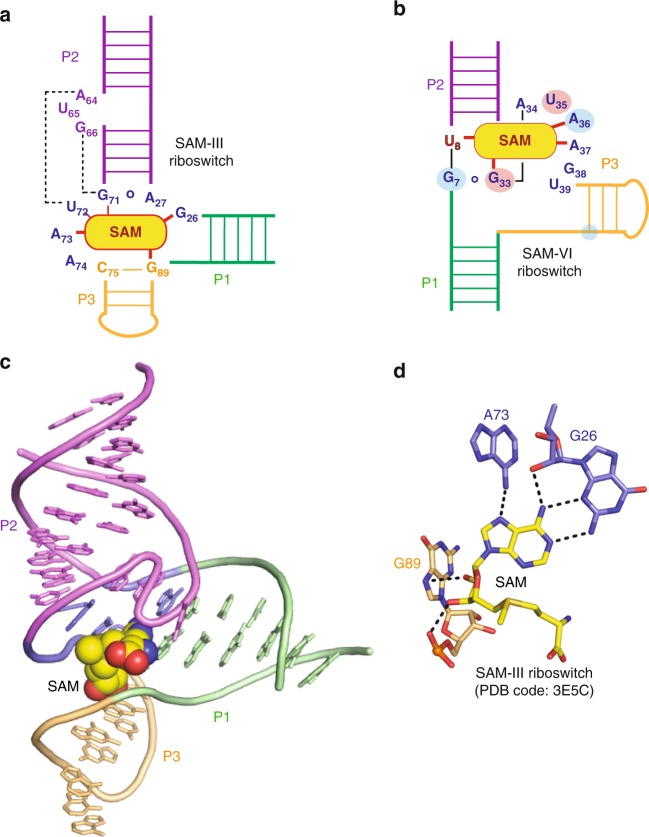
Comparison of secondary structures, tertiary folds, and binding pockets of SAM-III and SAM-VI riboswitches. **a, b** Secondary structure schemes of SAM-III (**a**) and SAM-VI (**b**); nucleotides crucial for the ligand recognition are assigned and direct interactions are highlighted by red lines. The residues in SAM-VI that are equivalent in interaction with the ligand compared to SAM-III are shown with light red (electrostatic interaction) and light blue (hydrogen-bonding interaction) shadow. Note that the same helix color code is applied for SAM-III and SAM-VI. **c** Cartoon representations of the tertiary structure of SAM-III bound to SAM (PDB code: 3E5C); helix colors are as in **a**. **d** Binding interactions of SAM ligand with the SAM-III RNA pocket. SAM-adenine adopts a *syn* conformation in SAM-III while it is *anti* in SAM-VI. SAM-adenine interacts with the sugar edge of a guanosine (G26) through its Watson-Crick edge in SAM-III while it is the Hoogsteen face of SAM-adenine that pairs with the Watson-Crick face of a uridine (U8) in SAM-VI.

The consensus sequence and secondary structure model of SAM-VI have been reported to share similarities with the SAM-III motif^13^. The SAM-VI crystal structure solved here allows a first comparison of the three-dimensional architectures of the two riboswitch classes^27^. Schematic drawings of their secondary structures and interactions with the ligand SAM—based on the crystal structures—are juxtaposed in Fig. 6a, b. Both SAM-III and SAM-VI adopt 3-way junctional folds. For SAM-III (Fig. 6a, c), stem P2 stacks pseudo-co-axially with stem P3, mediated by junctional nucleobase interactions at their interface; together, they form a long, slightly bent helix. Stem P1 is directed outwards from the junction, almost perpendicular towards stem P3. The ligand SAM is located in the junction and integrates from the major groove side of P3. Interestingly, the adenine of SAM is tilted towards the P2-P3 axis and becomes stacked between stems P2 and P1 (Fig. 6a, c). In contrast, for SAM-VI, stem P2 co-axially stacks with stem P1 (and not P3) to form a long helix, while stem P3 is perfectly perpendicular towards it (Figs. 1a, b and 6b). The ligand SAM is also located in the junction region, however, in SAM-VI, the SAM-adenine participates in the continuous base staple of the long helix (P1–P2) (Figs. 1d and 6b) and has no direct interaction with the stem perpendicular to its axis. Another major difference is that the bulge-junction interaction observed for SAM-III is not encountered in the SAM-VI fold (Fig. 6a, b).

Although in both SAM-III and SAM-VI riboswitches, the Watson-Crick and Hoogsteen faces of the SAM-adenine base are fully involved in hydrogen bonding, the individual recognition pattern through RNA nucleotides is very distinct (Figs. 2f and 6d). We furthermore point out that SAM binds in a different conformation to SAM-III compared to SAM-VI. First, the SAM-ribose adopts C2′-*endo* conformation in SAM-III (Fig. 6d) while it is C3′-*endo* in SAM-VI (Fig. 2f). Second, in SAM-III, the SAM-adenine base is in *syn* conformation at the glycosidic bond (Fig. 6d) while it is *anti* in SAM-VI (Fig. 2f).

In the SAM-III riboswitch, the adenine base of SAM interacts with the sugar edge of G26 through its Watson-Crick edge. Additionally, its Hoogsteen side (N7) is engaged in a hydrogen-bond with the NH6 of A73 (Fig. 6d). The sequence–equivalent residue of G26 (SAM-III) in SAM-VI riboswitch is G7. It does not hydrogen-bond with SAM, but pairs with G33 (SAM-VI) and stacks with the SAM-adenine (Figs. 2e and 6b). The sequence–equivalent residue of A73 (SAM-III) in SAM-VI is A36. It interacts with N1 of SAM-adenine, and not with N7 as in SAM-III (Figs. 2f and 6d). G89 (SAM-III) from stem P3 forms two hydrogen bonds with the ribose of SAM (Fig. 6d) while SAM-VI RNA recognizes the ligand’s ribose by a single H-bond only (between SAM 2′-OH and G33 phosphate) (Fig. 2f). A comparison of how the two SAM-riboswitch classes accommodate the reactive SAM sulfonium moiety shows that both seem to involve O4 groups of uridines (U72 in SAM-III Supplementary Fig. 4c and U6 in SAM-VI Fig. 2g). Although the distances are only in the 4 Å region, the vicinity of uridine O4 functionalities has been found also for other SAM riboswitches, most pronounced in the SAM-II motif^26^ indicating electrostatic interactions. In SAM-III, a second type of interaction between a ribose 2′-O (G71) and the sulfonium of SAM is observed (Supplementary Fig. 4). Taken together, our comparative analysis supports the notion that the fold and ligand recognition pattern of SAM-III and SAM-VI riboswitches are significantly distinct.

SAM-VI is the rarest riboswitch class discovered independently to date by using comparative sequence analysis (13). This example shows that only a few unique examples in a database (less than 20 for SAM-VI in Rfam database (RefSeq 80 dataset) and the accompanying lack of evidence for sequence and structural conservation should still encourage experimental testing of an otherwise poor riboswitch candidate, provided the sequence is associated with a gene contributing to metabolite biosynthesis (in the present case for a SAM synthetase) or transport. This feature is characteristic for all thus far known SAM riboswitches and made the discovery of the new class-VI possible (13). Support for SAM-VI and SAM-III as being independent classes also originated from the fact that organisms that carry SAM-VI (*Actinobacteria*) and SAM-III (*Firmicutes*) RNAs are phylogenetically distant. Our comprehensive structural study now unequivocally defines that SAM-III and SAM-VI folds and their recognition modes are distinct. Besides improving our general understanding of small molecule–RNA recognition, every single new SAM-riboswitch class with solved three-dimensional structure makes these scaffolds very attractive for efforts in engineering tools for chemical and synthetic biology. In particular, the structural diversity of synthetic SAM analogs that are already utilized in conjunction with protein enzymes (MTases) makes the engineering of (protein-free) SAM-riboswitch-based systems for new RNA labeling approaches highly promising.

## Methods

### RNA constructs design and sample preparation

Besides testing different length of stems P1 and P2, SAM-VI riboswitch constructs were designed with stable tetra-loop motifs UNCG and GNRA, as well as the U1A protein-binding loop at the position of the variable loop L2 to facilitate RNA crystallization. These sequences adjoined by the HDV ribozyme sequence were inserted into a PUT7 vector^50^, which was amplified in *Escherichia coli* DH5α cells using the ZQZY-CF incubator shaker (Shanghai Zhichu Instrument) at 37 °C for 12 h, and after lysis and purification by PureLink™ Expi Endotoxin-Free Mega Plasmid Purification Kit (Invitrogen^TM^, A31232), cleaved with *Hind* III restriction enzyme. The linearized DNA templates were transcribed in vitro by T7 RNA polymerase. The RNA was purified using denaturing polyacrylamide gel electrophoresis (PAGE). The full-length product was visualized under a UV lamp, excised and electro-eluted by the Elutrap electro-elution system (GE Healthcare) into 0.5 × Tris-acetate-EDTA (TAE) buffer at 4 °C. The eluted RNA sample was then precipitated with isopropanol and washed with 70% ethanol, which was followed by lyophilisation. Finally, the lyophilized RNA was dissolved in diethyl pyrocarbonate (DEPC) treated, double-distilled water.

### U1A protein expression and purification

The U1A (2-98) Y31H/Q36R protein with an N-terminal His10-SUMO tag and an ubiquitin-like protease (ULP1) cleavage site was expressed in *Escherichia coli* (BL21-Condon plus strain). Cells were resuspended in buffer A (25 mM Tris, pH 8.0, 500 mM NaCl, 5 mM 2-mercaptoethanol (β-ME), 5 mM imidazole) supplemented with 0.1 mM phenylmethylsulfonylfluorid (PMSF). After cell disruption and centrifugation, the supernatant was loaded to a buffer A pre-equilibrated HisTrap column (GE Healthcare). After washing with 25 column volumes of buffer A, the fusion protein was eluted with buffer B (same as buffer A, but 500 mM instead of 5 mM imidazole). The His10-SUMO tag was removed by ULP1 cleavage and separated from U1A by re-loading onto the second HisTrap column. Protein was further purified by chromatography using a HiTrap Heparin SP column (GE Healthcare), followed by chromatography on a HiLoad Superdex 75 16/60 column (GE Healthcare). Purified U1A was then concentrated up to 10 mg/ml in buffer C (40 mM HEPES, pH 7.0, 100 mM NaCl, 50 mM KCl, 10 mM DTT) for further experiments. For the expression of selenomethionine (Se-Met) derivative U1A protein, cells were grown in M9 medium supplemented with Se-Met and the other amino acids. The Se-Met U1A protein was purified by the same method as described above.

### Ligand synthesis

SAM and SAH were purchased from Sigma. The *N*-mustard analog of *S*-adenosyl-L-methionine (chemical structure of M1 shown in Fig. 3d) was chemically synthesized in nine steps each in analogy to reports in the literature^43–45^. NMR spectroscopic and mass spectrometric data of M1 are shown in Supplementary Figs. 9–13.

### Crystallization and structure determination

A final concentration of 0.25 mM *Bifidobacterium* SAM-VI riboswitch RNA in 40 mM HEPES, pH 7.4, 52 mM KCl, 5 mM MgCl_2_ was annealed at 65 °C for 5 min and cooled on ice for half an hour, which was followed by addition with SAM or SAH or other ligands to a final concentration of 3 mM, and U1A protein to a final concentration of 0.375 mM. Crystallization were setup at 16 °C by mixing 0.2 μl of the RNA-ligand complex with the reservoir solution at an equimolar ratio using sitting drop vapour diffusion method with the ARI Grphon-LCP-Nano robot. Well-diffracted crystals were grown from the condition comprising 0.1 M sodium acetate trihydrate pH 4.6, 8-12% w/v polyethylene glycol 4000 over a period of one week. The crystals were transferred in mother liquor supplemented with 20% glycerol and flash frozen in liquid nitrogen. X-ray diffraction data were collected on beamline BL17U1 at the Shanghai Synchrotron Radiation Facility (SSRF). The diffraction data were processed using HKL2000 (HKL Research). The phase problem was solved with the single-wavelength anomalous diffraction (SAD) method, in which anomalous data was collected from the atom selenium that was introduced by using Se-Met derivative U1A protein in co-crystallization. The selenium atoms were located with the Autosol program in the Phenix suite^51^. The model was further built and refined using Coot^52^, Refmac^53^ and Phenix programs^51^. Crystal diffraction data and refinement statistics are shown in Supplementary Table 1.

Finally, we added the ligand SAM to our structure in the last several runs of refinement. The 2 fo-fc electron-density of SAM is shown in Fig. 2f. The overall electron density of the SAM-VI riboswitch molecule is shown in Supplementary Fig. 2a.

### Isothermal titration calorimetry

All ITC experiments were performed on a MicroCal PEAQ-ITC calorimeter at 20 °C. Prior to titration, 0.05-0.1 mM *Bifidobacterium* SAM-VI riboswitch RNA was dialysed overnight at room temperature against ITC buffer containing 40 mM HEPES, pH 7.0, 50 mM KCl, 10 mM MgCl_2_. RNAs were refolded at 65 °C for 5 min and cooled on ice before titration. The ligands were dissolved in the dialysis buffer at the concentration of 0.5-1 mM and injected into the sample cell that was filled with 203 μl of RNA sample in a volume of a single initial injection of 1 µl, followed by 18 injections of 2 µl ligand into RNA sample, with a 0.5 μl s^−1^ rate, 120 s intervals between injections and a reference power of 5 μcal s^−1^. Integrated heat data were analyzed using a one-site binding model via MicroCal PEAQ-ITC Analysis Software, provided by the manufacturer. All ITC titration experiments were independently repeated (three replicates in total) and the complete sets of thermodynamic binding parameters are provided in Supplementary Table 2.

### Steady-state fluorescence spectroscopy

All steady-state fluorescence spectroscopic experiments were measured on a Cary Eclipse spectrometer (Varian, Australia) equipped with a peltier block, a magnetic stirring device, and a RX2000 stopped-flow apparatus (Applied Photophysics Ltd., UK). The data obtained were processed with OriginPro 2018 software (OriginLab, USA).

### Binding affinities

Ap-modified RNA samples were prepared in 0.5 μM concentration in a total volume of 1 mL of buffer (50 mM KMOPS pH 7.5, 100 mM KCl, 2 mM MgCl_2_). The samples were heated to 90 °C for 2 min, allowed to cool to room temperature, and held at 20 °C in the peltier controlled sample holder. Then, ligands were manually pipetted in a way not to exceed a total volume increase of 2%. The solution was stirred during each titration step and allowed to equilibrate for at least 15 min before data collection. Spectra were recorded from 330 to 450 nm using the following instrumental parameters: excitation wavelength, 308 nm; increments, 1 nm; scan rate, 120 nm/min; slit widths, 10 nm. The apparent binding constants *K*_d_ were determined by following the increase in fluorescence after each titration step via integration of the area between 330 and 450 nm. Changes in fluorescence (*F*—*F*_0_) were normalized to the maximum fluorescence measured at the maximum concentration of ligand. The measurement for each titration step was repeated at least three times and the mean of the normalized fluorescence intensity and the corresponding error bars for each value were plotted against the ligand concentration. Data were fit using a two-parametric (*K*_d_ and *δ*) quadratic Equation (1) implying 1:1 stoichiometry:

1 $$\frac{{F - F_0}}{{F_{\rm{f}} - F_0}} = \frac{{K_{\rm{d}} + [{\mathrm{Ligand}}]{\mathrm{tot}} + [{\mathrm{RNA}}]{\rm{tot}} + \sqrt {(K_{\rm{d}} + [{\mathrm{Ligand}}]{\mathrm{tot}} + [{\mathrm{RNA}}]{\rm{tot}})^2 - 4[{\mathrm{Ligand}}]{\mathrm{tot}} \cdot [{\mathrm{RNA}}]{\rm{tot}}} }}{{2([{\mathrm{RNA}}]{\rm{tot}} - \delta )}}$$

where *F*_0_ corresponds to initial fluorescence; *F*_f_ corresponds to final fluorescence; [RNA]_tot_ is the total Ap-RNA concentration; [Ligand]_tot_ is the total ligand concentration in the sample for each titration step. The parameter *δ* was introduced for correct normalizing of the data in cases when saturation of a RNA by a ligand cannot be reached. The final *K*_d_ value is determined from fitting of data obtained from three independent titration experiments.

The standard deviation corresponding to each value of the normalized fluorescence intensity were calculated using Equation (2):

2 $${\mathrm{Error}}\left( {\frac{{F - F_0}}{{F_{\rm{f}} - F_0}}} \right) = \frac{{\sqrt {({\mathrm{SF}})^2 + \left( {\frac{{F - F_{\rm{f}}}}{{F_{\rm{f}} - F_0}} \cdot {\mathrm{SF}}_0} \right)^2 + \left( {\frac{{F - F_0}}{{F_{\rm{f}} - F_0}} \cdot {\mathrm{SF}}_{\rm{f}}} \right)^2} }}{{F_{\rm{f}} - F_0}}$$

where SF corresponds to the standard error of the mean (SEM) of fluorescence intensity for each titration step, SF_0_ and SF_f_ correspond to the SEM of initial and final fluorescence intensities, respectively.

### Rate constants

Observed rate constants *k*’ for individual riboswitch variants (wt U39Ap and U6C U39Ap) were measured under pseudo-first-order conditions with a ligand in excess over RNA. Stock solutions were prepared for each Ap variant (concentration *C*_RNA_ = 0.6 μM in 50 mM KMOPS pH 7.5, 100 mM KCl, 2 mM MgCl_2_) and for ligand (concentration *C*_Ligand_ = 0.9–45 μM in 50 mM KMOPS pH 7.5, 100 mM KCl, 2 mM MgCl_2_). Mixing equal volumes of these stock solutions via the stopped-flow apparatus resulted in a final concentration of 0.3 μM for RNA and of 0.45–22.5 μM for ligand. Spectra were recorded at 20 °C using the following instrumental parameters for the Ap variants: excitation wavelength, 308 nm; emission wavelength, 372 nm; increment of data point collection, 0.2 s; slit widths, 10 nm.

The stopped-flow fluorescence data were fit using a three-parameter (*A*_1_, *A*_2_, and *k*’) single-exponential Equation (3) for 1:1 stoichiometry:

3 $$F = A_1 + A_2 \cdot {\mathrm{e}}^{ - k^\prime \cdot t}$$

*A*_1_ final fluorescence

*A*_2_·e^−*k*′*·t*^change in fluorescence over time (*t*) at the observed rate *k*′.

The measurement for each concentration was repeated at least three times, then the mean values of the observed rates *k*′, and the corresponding error bars were plotted against concentration of a ligand to obtain the on-rate constant *k*_on_ (also *k*_293K_) from the slope of the plot.

### LacZ reporter assays

The wild-type (WT) and mutant sequences (M1, M2, M3) of *B. angulatum* 59 *metK* SAM-VI riboswitch were amplified by PCR and cloned into the vector pUCm-T (Sangon Biotech, Shanghai) upstream of the *E.coli lacZ* gene (Fig. 5d). DH5α strains carrying the riboswitch reporter construct were cultured at 37 °C for 8-10 h in the presence of ampicillin, with or without SAM. X-gal was added to the cultured cell with final concentration of 200 μg/mL for visual detection of the reporter gene expression. In quantitative β-galactosidase assays, 80 μl of cultured cells were transferred to each well of tissue culture 96-well plates. Absorbance at 595 nm was measured on a Synergy NEO2 Hybrid Multi-Mode Reader. Then, 80 μl of Z buffer (60 mM Na_2_HPO_4_, 40 mM NaH_2_PO_4_, 10 mM KCl, 1 mM MgSO_4_) and 40 μl of a 1 mg ml^−1^ 4-methylumbelliferyl-β-D-galactopyranoside aqueous solution (4-MUG, Sangon Biotech, Shanghai) were added to each well and incubated at room temperature for 15 min before adding 40 μl of a 1 M Na_2_CO_3_ aqueous solution to quench the reaction. Fluorescence was measured at 360 nm excitation and 460 nm emission. The average relative fluorescence values were calculated as previously reported^54^. All assays were performed in triplicate.

### Reporting summary

Further information on research design is available in the Nature Research Reporting Summary linked to this Article.

## Supplementary information

Supplementary Information

Reporting Summary

## Data Availability

The data supporting this study are available from the corresponding authors upon reasonable request. The atomic coordinates and structure factors for the reported crystal structures of SAM-VI riboswitch have been deposited to the Protein Data bank under accession number 6LAS (bound to SAM), 6LAU (bound to SAH), 6LAZ (bound to M1) and 6LAX (U6C mutant bound to SAM).

## Source data

Source Data
