# Supplementary material for: SAM-VI riboswitch structure and signature for ligand discrimination
Source: Nat Commun. 2019 Dec 16;10:5728. doi: 10.1038/s41467-019-13600-9 (PMC6914780; doi:10.1038/s41467-019-13600-9)
Supplement: Supplementary file 1 — Supplementary Information [file 41467_2019_13600_MOESM1_ESM.pdf]

# Supplementary Information

## SAM-VI riboswitch structure and signature for ligand discrimination

**Aiai Sun[1,#], Catherina Gasser[2,#], Fudong Li[3,#], Hao Chen[1],**

**Stefan Mair[2], Olga Krasheninina[2], Ronald Micura[2,*], and Aiming Ren[1,*]**

[1]Life Science Institute, Zhejiang University,

Hangzhou, Zhejiang 310058, China

[2]Institute of Organic Chemistry, Center for Molecular Biosciences Innsbruck,

Leopold Franzens University, Innsbruck, A6020, Austria

[3]Hefei National Laboratory for Physical Sciences at the Microscale, School of Life Sciences, University of Science and Technology of China, 230026 Hefei, China

* Corresponding authors: aimingren@zju.edu.cn (AR) and ronald.micura@uibk.ac.at (RM)

[#]These authors contributed equally.

# Contents


| | |
|---|---|
| **Supplementary Table 1** | 3 |
| **Supplementary Table 2** | 4 |
| **Supplementary Table 3** | 5 |
| | |
| **Supplementary Figure 1** | 6 |
| **Supplementary Figure 2** | 7 |
| **Supplementary Figure 3** | 8 |
| **Supplementary Figure 4** | 9 |
| **Supplementary Figure 5** | 10 |
| **Supplementary Figure 6** | 11 |
| **Supplementary Figure 7** | 12 |
| **Supplementary Figure 8** | 13 |
| **Supplementary Figure 9** | 14 |
| **Supplementary Figure 10** | 15 |
| **Supplementary Figure 11** | 16 |
| **Supplementary Figure 12** | 17 |
| **Supplementary Figure 13** | 18 |
| **Supplementary Figure 14** | 19 |
| **Supplementary Figure 15** | 20 |
| **Supplementary Figure 16** | 21 |
| **Supplementary Figure 17** | 22 |
| **Supplementary Figure 18** | 23 |
| **Supplementary Figure 19** | 24 |
| **Supplementary Figure 20** | 25 |
| **Supplementary Figure 21** | 26 |
| **Supplementary Figure 22** | 27 |

**Supplementary Information**

**Supplementary Table 1 | Crystallographic statistics for the *B. angulatum* SAM-VI riboswitches bound to SAM, SAH, or ligand mimic M1.**

| | SAM-VI Bound to SAM | SAM-VI Bound to SAH | SAM-VI Bound to M1 | SAM-VI U6C Bound to SAM |
|---|---|---|---|---|
| **Data collection** | | | | |
| Space group | $P\ 2_1$ | $P\ 2_1$ | $P\ 2_1$ | $P\ 2_1$ |
| Cell dimensions | | | | |
| a, b, c (Å) | 56.4, 84.2, 90.5 | 56.1, 85.0, 93.4 | 56.4, 85.4, 93.2 | 45.9, 86.9, 93.6 |
| α, β, γ (°) | 90, 105.8, 90 | 90, 105.8, 90 | 90, 105.5, 90 | 90, 99.2, 90 |
| Wavelength (Å) | 0.979 | 1.102 | 1.102 | 0.979 |
| Resolution (Å) | 40.0-2.7 (2.75-2.7) | 40.0-3.1 (3.15-3.1) | 42.7-2.8 (2.9-2.8) | 50.0-2.7 (2.75-2.7) |
| $R_{pim}$ (%) | 5.6(42.3) | 6.1(49.2) | 3.1(28.5) | 6.8(43.5) |
| $I / \sigma I$ | 12.1(1.2) | 8.7(1.0) | 15.9(2.6) | 10.2(1.3) |
| Completeness (%) | 99.7(99.9) | 97.8(91.5) | 94.8(95.4) | 97.5(99.5) |
| Redundancy | 6.7(6.8) | 6.7(6.0) | 6.9(6.9) | 5.2(5.3) |
| CC(1/2) | 0.98(0.68) | 1.00(0.67) | 1.00(0.89) | 1.00(0.65) |
| **Refinement** | | | | |
| No. reflections | 22135 | 14883 | 20945 | 19588 |
| $R_{work}$ / $R_{free}$ (%) | 20.6/24.4 | 19.1/25.1 | 18.8/22.7 | 18.9/23.9 |
| No. of atoms | | | | |
| Protein | 2169 | 2159 | 2176 | 2217 |
| RNA | 2352 | 2329 | 2368 | 2352 |
| Ligand | 54 | 52 | 58 | 54 |
| Water | 22 | 33 | 21 | 38 |
| *B*-factors (Å$^2$) | | | | |
| Protein | 45.1 | 56.8 | 65.9 | 37.4 |
| RNA | 62.4 | 94.9 | 91.8 | 55.7 |
| Ligand | 54.3 | 88.3 | 86.4 | 47.0 |
| Water | 47.6 | 50.4 | 66.2 | 30.3 |
| R.M.S. deviations | | | | |
| Bond length (Å) | 0.008 | 0.010 | 0.008 | 0.009 |
| Bond angles (°) | 1.2 | 1.4 | 1.3 | 1.2 |
| Ramachandran values (%) | | | | |
| Most favored | 97.0 | 94.3 | 95.5 | 94.9 |
| Additional allowed | 3.0 | 5.7 | 4.5 | 5.1 |
| Outliers | 0 | 0.4 | 0 | 0 |

*Values in parentheses are for highest-resolution shell.

**Supplementary Table 2 | Thermodynamic parameters of ligand binding to SAM-VI RNAs determined by isothermal titration calorimetry (ITC).**

| RNA | Ligand | ΔH | -TΔS | ΔG | ΔΔG | N[a] | $K_d$[b] [μM] | c-value | $K_d$[c] (Mean) [μM] |
|---|---|---|---|---|---|---|---|---|---|
| | | [kcal/mol] | | | [kcal/mol] | | | | |
| SAM-VI WT | SAM | -32.3±0.2 | 23.6 | -8.8 | - | 0.9 | 0.28±0.01 | 122.0 | 0.33±0.06 |
| | | -32.7±0.2 | 24.1 | -8.7 | | 0.9 | 0.33±0.02 | 102.8 | |
| | | -31.3±0.2 | 22.6 | -8.6 | | 0.9 | 0.39±0.02 | 138.4 | |
| | SAH | -33.5±2.0 | 26.8 | -6.7 | 2.0 | 0.5 | 10.0±1.1 | 4.0 | 10.9±1.3 |
| | | -33.5±1.3 | 26.8 | -6.6 | | 0.7 | 12.4±1.0 | 3.9 | |
| | | -32.3±1.9 | 25.6 | -6.7 | | 0.5 | 10.3±1.1 | 3.1 | |
| | M1 | -24.4±0.5 | 17.3 | -7.2 | 1.5 | 0.7 | 4.3±0.3 | 9.2 | 4.4±0.5 |
| | | -25.1±0.1 | 17.9 | -7.3 | | 1.1 | 3.9±0.1 | 8.2 | |
| | | -23.9±0.3 | 16.8 | -7.1 | | 0.7 | 4.9±0.2 | 10.4 | |
| SAM-VI U6C | SAM | -28.9±0.5 | 20.5 | -8.5 | 0.2 | 0.9 | 0.45±0.07 | 88.3 | 0.46±0.02 |
| | | -29.2±0.3 | 20.7 | -8.5 | | 1.1 | 0.48±0.05 | 86.8 | |
| | | -28.7±0.3 | 20.1 | -8.5 | | 1.0 | 0.46±0.04 | 84.2 | |
| | SAH | -29.6±0.4 | 22.2 | -7.6 | 1.1 | 0.8 | 2.4±0.2 | 18.0 | 2.3±0.1 |
| | | -27.7±0.1 | 20.2 | -7.6 | | 1.0 | 2.2±0.1 | 16.9 | |
| | | -27.5±0.2 | 20.0 | -7.6 | | 1.0 | 2.2±0.1 | 17.9 | |
| | M1 | -22.0±0.2 | 14.4 | -7.6 | 0.9 | 1.1 | 2.0±0.1 | 19.8 | 1.7±0.3 |
| | | -20.7±0.5 | 12.9 | -7.8 | | 1.4 | 1.5±0.2 | 26.0 | |
| | | -20.3±0.2 | 12.5 | -7.8 | | 1.2 | 1.5±0.1 | 27.0 | |
| SAM-VI G7C | SAM | | | | | | N.D.[d] | | |
| SAM-VI U8C | SAM | | | | | | N.D. | | |
| SAM-VI U8G | SAM | | | | | | N.D. | | |
| SAM-VI U8A | SAM | | | | | | N.D. | | |
| SAM-VI G33A | SAM | | | | | | N.D. | | |
| SAM-VI A34C | SAM | | | | | | N.D. | | |
| SAM-VI A34G | SAM | | | | | | N.D. | | |
| SAM-VI A36C | SAM | | | | | | N.D. | | |
| SAM-VI A37C | SAM | | | | | | N.D. | | |
| SAM-VI G38C | SAM | | | | | | N.D. | | |
| SAM-VI A36G/A37G/G38A | SAM | | | | | | N.D. | | |

[a] N refers to the stoichiometric ratio of ligand to RNA.
[b] The fitting binding disassociation parameter of each independent titration.
[c] Reported error is the standard deviation of three independent experiments.
[d] N.D., No detectable interaction under the experiment conditions.

**Supplementary Table 3 | Thermodynamic and kinetic parameters of ligand binding to SAM-VI and comparison to other SAM riboswitch classes.**

| **SAM riboswitch class**<br>Ligand | $K_d$ 293 K<br>[μM] | $k_{on}$ 293 K<br>[$M^{-1}s^{-1}$] |
|---|---|---|
| **SAM-VI** | | |
| SAM | 0.33 ± 0.06 | 24580 ± 689 |
| SAH | 10.9 ± 1.3 | 5500 ± 266 |
| Ligand M1 | 4.4 ± 0.5 | 17240 ± 1140 |
| | | |
| **U6C SAM-VI** | | |
| SAM | 0.46 ± 0.02 | 62550 ± 1900 |
| SAH | 2.3 ± 0.1 | 1460 ± 179 |
| Ligand M1 | 1.7 ± 0.3 | 4230 ± 462 |
| | | |
| **SAM-III** | | |
| SAM | 0.85 [1] | 110000 [1] |
| | | |
| **SAM-II** | | |
| SAM | 0.200 ± 0.002 [2] | 9410 ± 1600 [2] |
| SAH | >1000[3] | |
| | | |
| **SAM-I** | | |
| SAM | 0.004-0.005 [3,4] | |
| SAH | 0.400 [3,4] | |
| | | |
| | | |

[1] Values reported in Smith, A.M. et al., **2010**. The SAM-responsive S-MK box is a reversible riboswitch. *Mol. Microbiol.*, 78(6), pp.1393–1402.

[2] Values reported in Haller, A. et al., **2011**. Conformational capture of the SAM-II riboswitch. *Nat. Chem. Biol.*, 7(6), pp.393–400.

[3] Values reported in Lim, J. et al., **2006**. Molecular-Recognition Characteristics of SAM-Binding Riboswitches. *Angew. Chem. Int. Ed.*, 45(6), pp.964–968.

[4] Values reported in Winkler, W.C., et al., **2003**. An mRNA structure that controls gene expression by binding *S*-adenosylmethionine. *Nat. Struct. Biol.*, **10**(9), pp. 701-707.

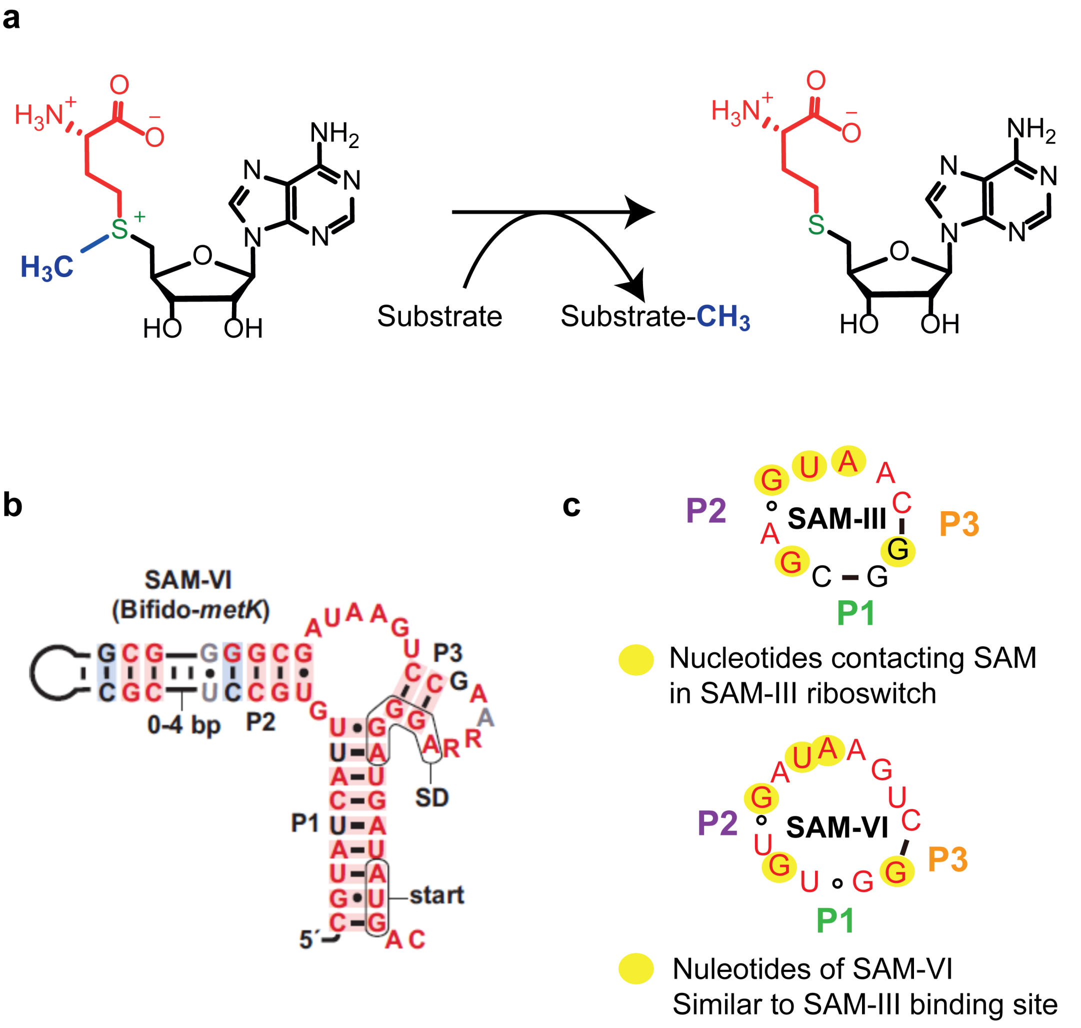


**Supplementary Figure 1 | The SAM-VI riboswitch class. a**, Chemical structure of SAM and SAH; methyl transfer reactions are catalysed by methyl transferases (MTases) and represent a major application of this cofactor system in living systems. **b,** Consensus sequence and secondary structure model of the SAM-VI riboswitch (*Bifido*-metK) revealed by the Breaker group (Mirihana et.al, *RNA biology*, 2018, 15(3):371-378.). **c**, Several nucleotides in the SAM-VI three-way junction (top, shaded in yellow) seem to have similar identity and location to ligand-contacting nucleotides in the SAM-III pocket. The nucleotides similar for SAM-VI and those in a representative SAM-III RNA that directly contact the ligand are shaded in yellow (bottom).

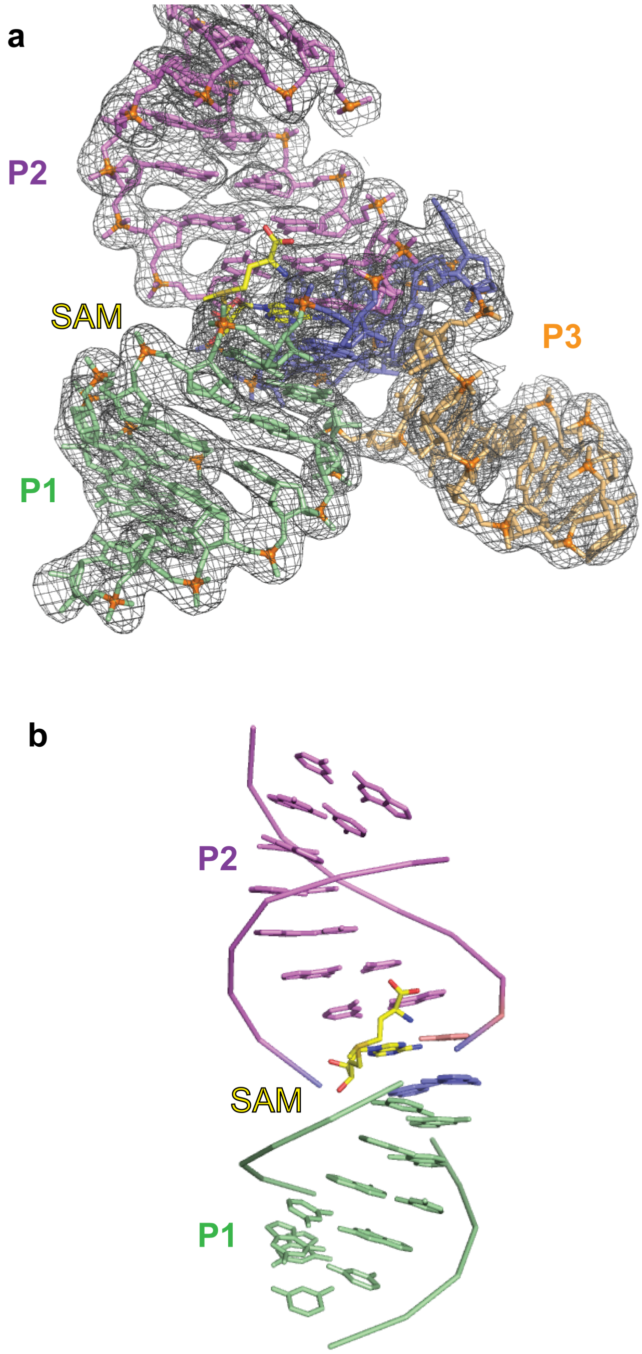


**Supplementary Figure 2 | SAM-VI riboswitch in complex with SAM. a**, The final, refined 2$F_o$-$F_c$ electron-density map contoured at 1.0 σ level with the built model of SAM-VI riboswitch is shown; for clarity, the electron-density of the U1A protein and the RNA binding site was omitted. **b**, Continuous stacking of canonical and junctional segments in the tertiary structure of the SAM-VI riboswitch bound to SAM.

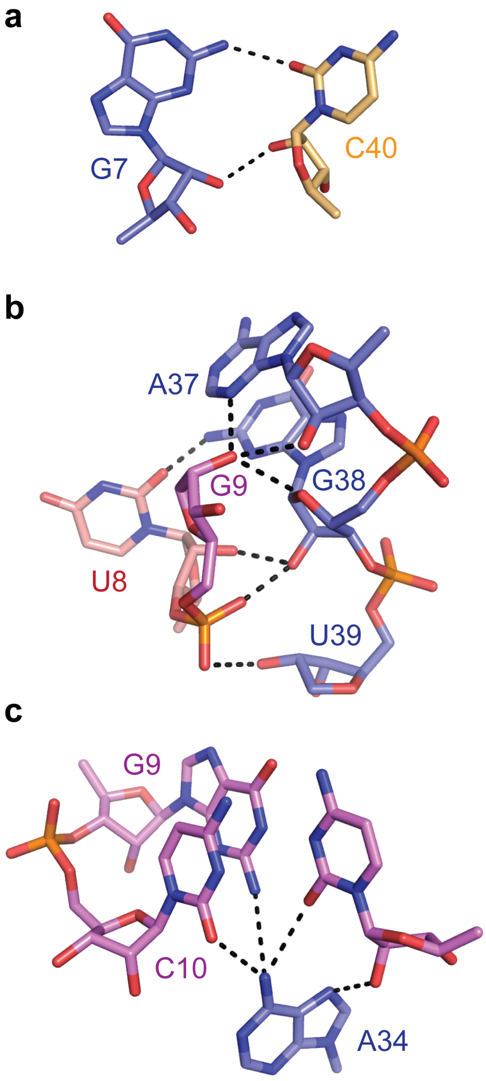


**Supplementary Figure 3 | Details of nucleotide interactions in the SAM-VI riboswitch in complex with SAM.** For discussion see the main text.

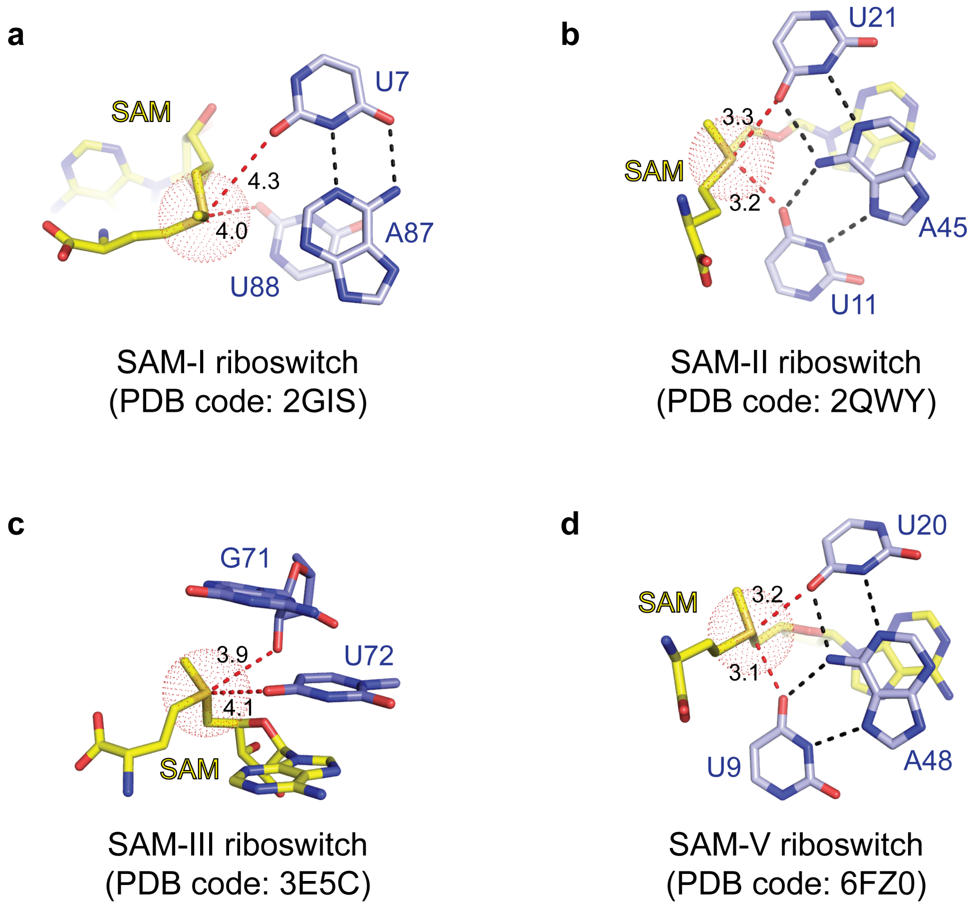


**Supplementary Figure 4 | Electrostatic interactions of the sulfonium moiety of SAM in SAM-I (a), SAM-II (b), SAM-III (c) and SAM-V (d).** The sulfonium moiety of SAM was shown with a ball of red dots. The electrostatic interactions were highlighted with red dash line and the distances to the electrostatic-interaction-involved oxygen were labeled along the red dash line.

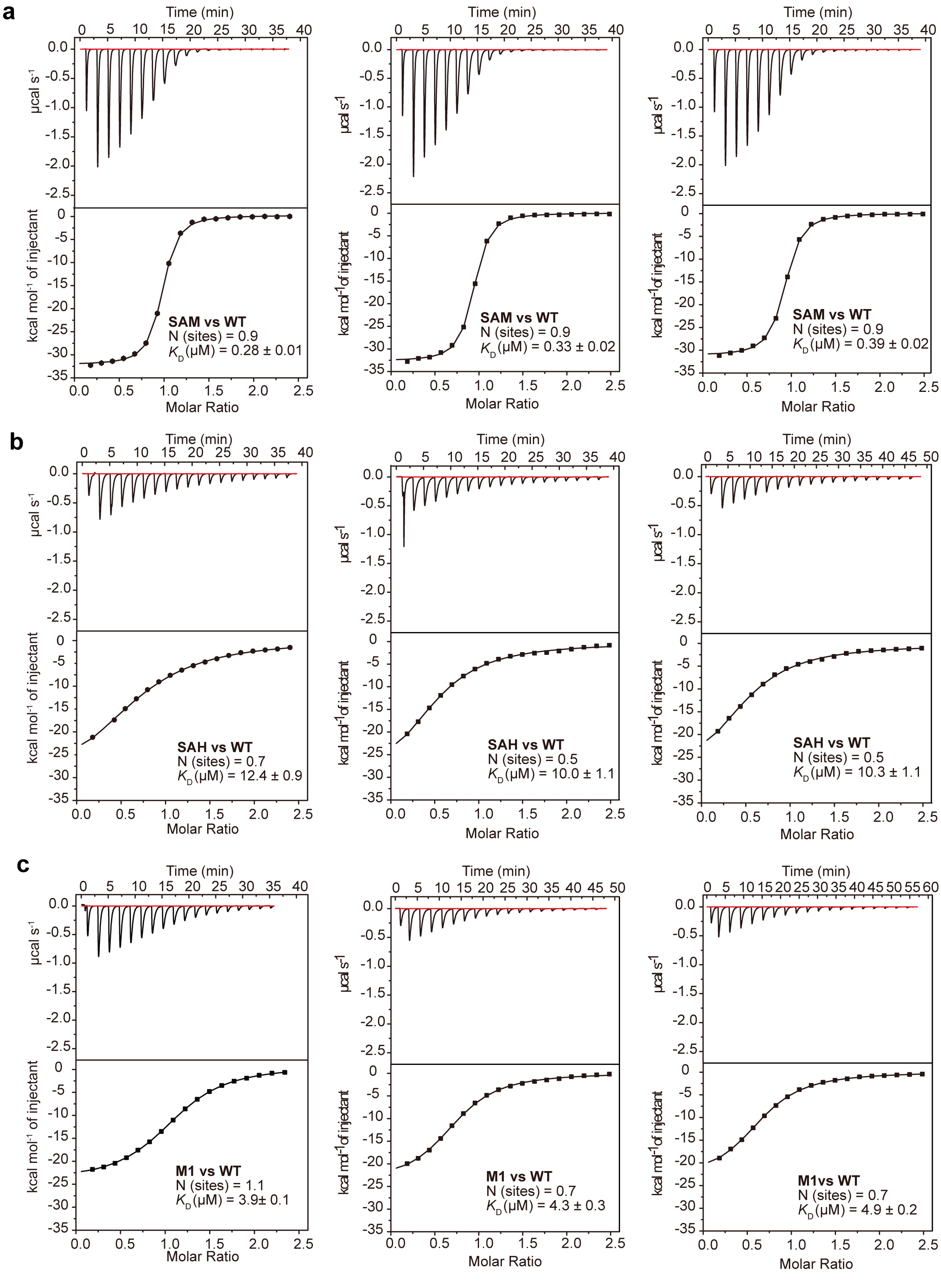


**Supplementary Figure 5 | Three independently repeated ITC titration experiments of SAM-VI riboswitch binding with SAM (a), SAH (b) and M1 (c).** All titrations were performed at 20 °C in 40 mM HEPES, pH 7.0, 50 mM KCl, 10 mM $MgCl_2$. For c-values see Supplementary Table 2. The $K_d$ and N parameters obtained from the fits are shown as text in the individual ΔH windows.

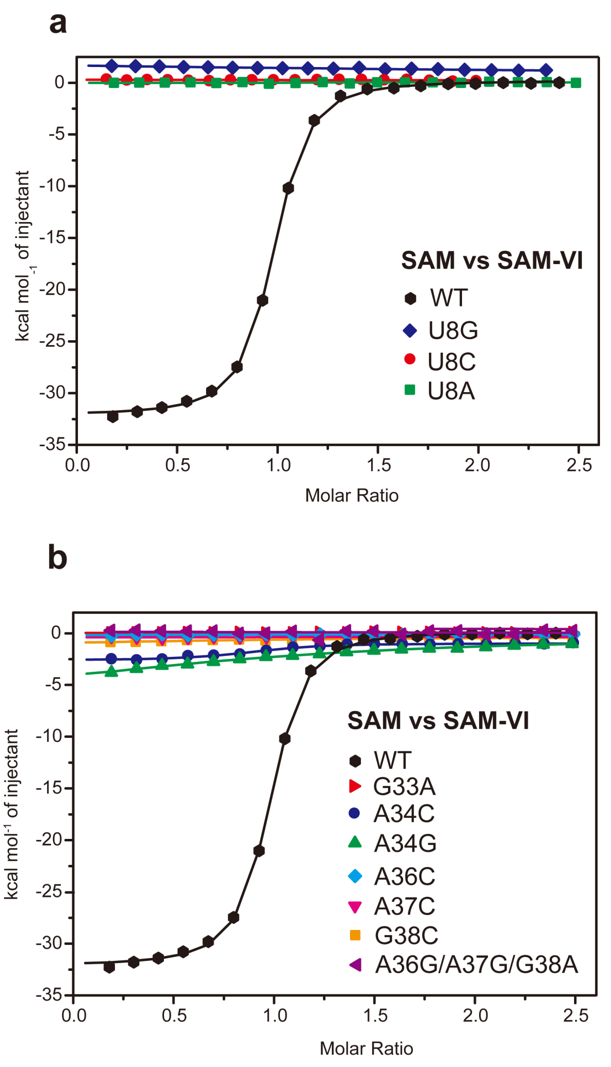


**Supplementary Figure 6 | Integrated fitted heat plots of SAM binding with SAM-VI riboswitch and mutants.** ITC measurements for U8 SAM-VI mutants (**a**) and G33, A34, A36 to G38 SAM-VI mutants (**b**) were performed at 20 °C in 40 mM HEPES, pH 7.0, 50 mM KCl, 10 mM $MgCl_2$. The SAM-VI riboswitch was in the measuring cell at 0.05-0.1 mM concentration; ligand was in the syringe at a concentration that was 10-fold higher than the RNA.

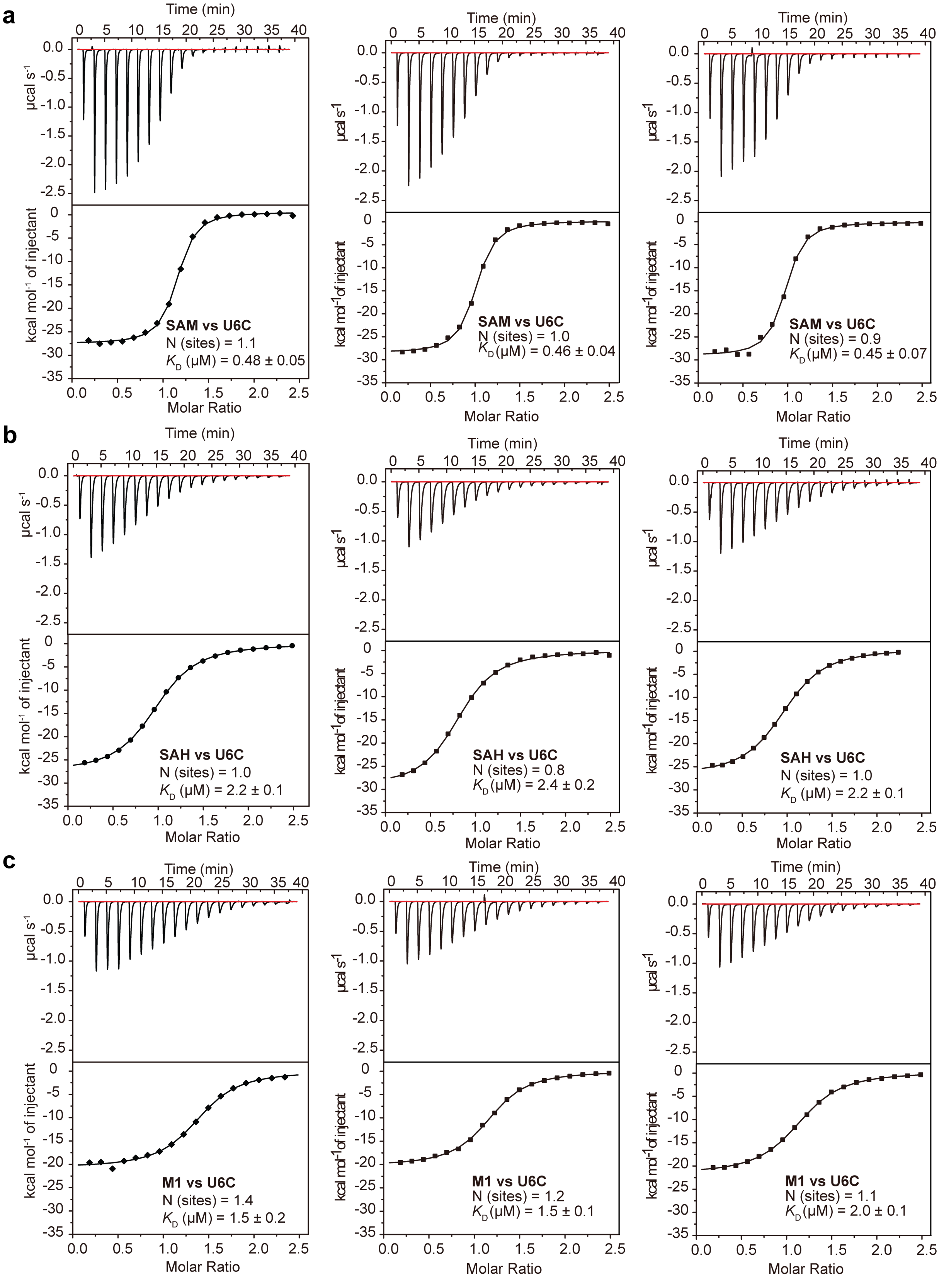


**Supplementary Figure 7 | Three independently repeated ITC titration experiments of SAM-VI riboswitch U6C mutant binding with SAM (a), SAH (b) and M1 (c).** All titrations were performed at 20 °C in 40 mM HEPES, pH 7.0, 50 mM KCl, 10 mM $MgCl_2$. For c-values see Supplementary Table 2. The $K_d$ and N parameters obtained from the fits are shown as text in the individual ΔH windows.

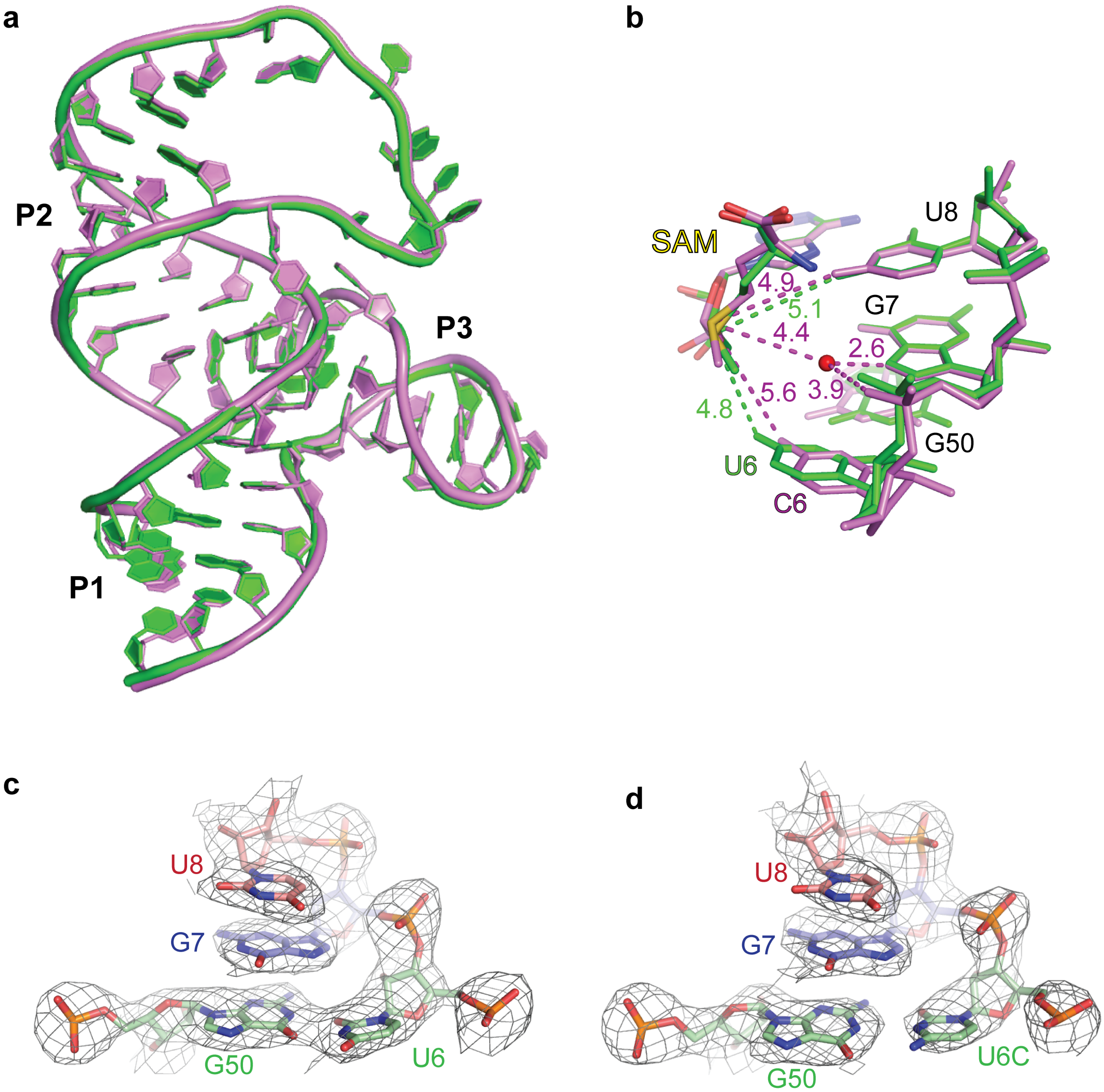


**Supplementary Figure 8 | Comparison of the wild-type (WT) SAM-VI riboswitch and the U6C mutant structures.** **a**, Superposition of the overall folds of wild-type SAM-VI riboswitch (in green) and U6C mutant (in violet). **b**, Superposition of key residues in wild-type SAM-VI riboswitch (in green) and U6C mutant (in violet) to visualize distinctions in interactions between the sulfonium group of SAM and its recognition site. **c-d**, Composite omit electron-density maps of the key residues for sulfonium recognition of wild-type SAM-VI riboswitch (**c**) and U6C mutant (**d**) contoured at 1.0 σ level.

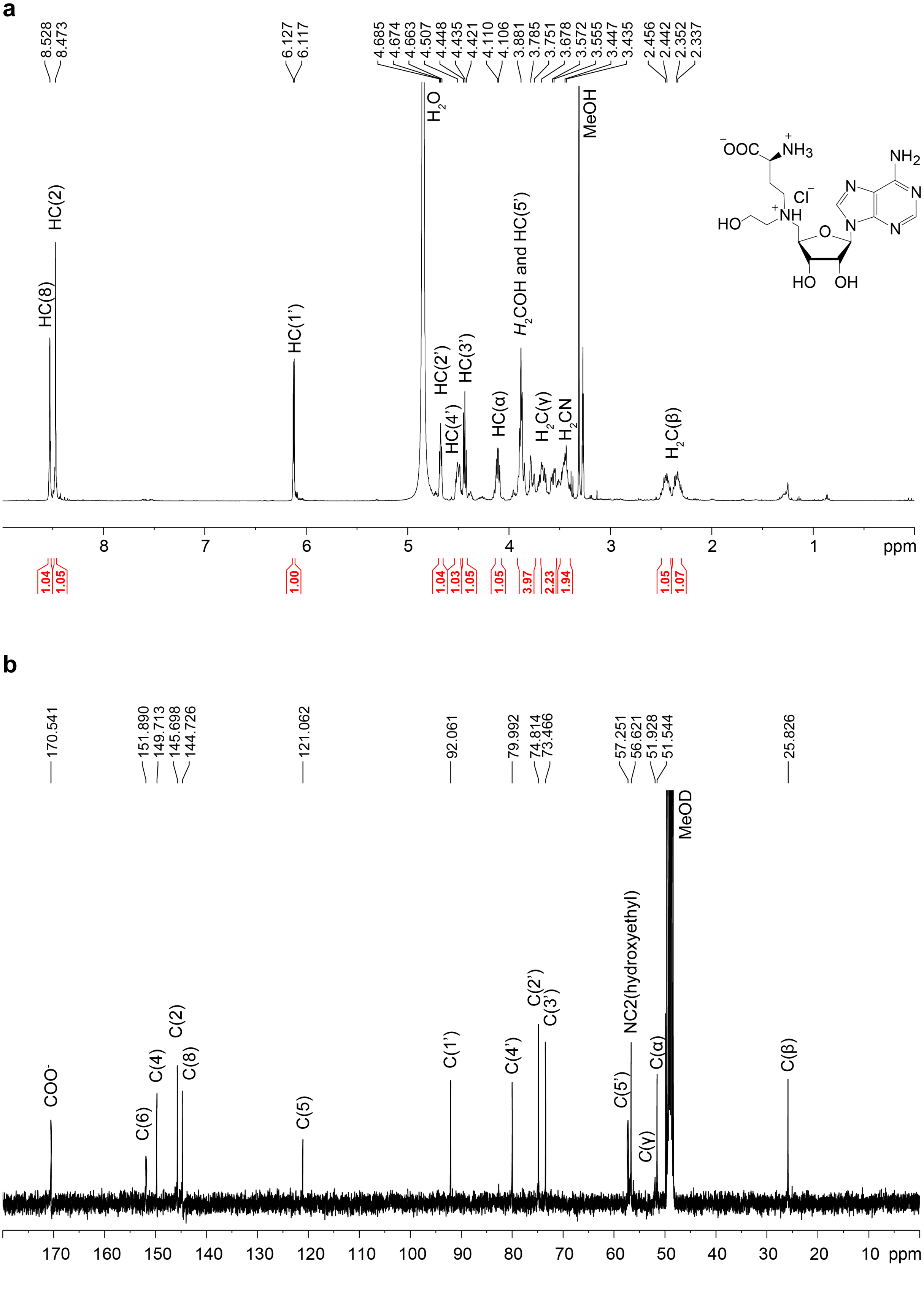


**Supplementary Figure 9 | NMR spectra of ligand analog M1. a**, $^{1}H$ NMR (400 MHz, MeOD) spectrum. **b**, $^{13}C$ NMR (100 MHz, MeOD) spectrum.

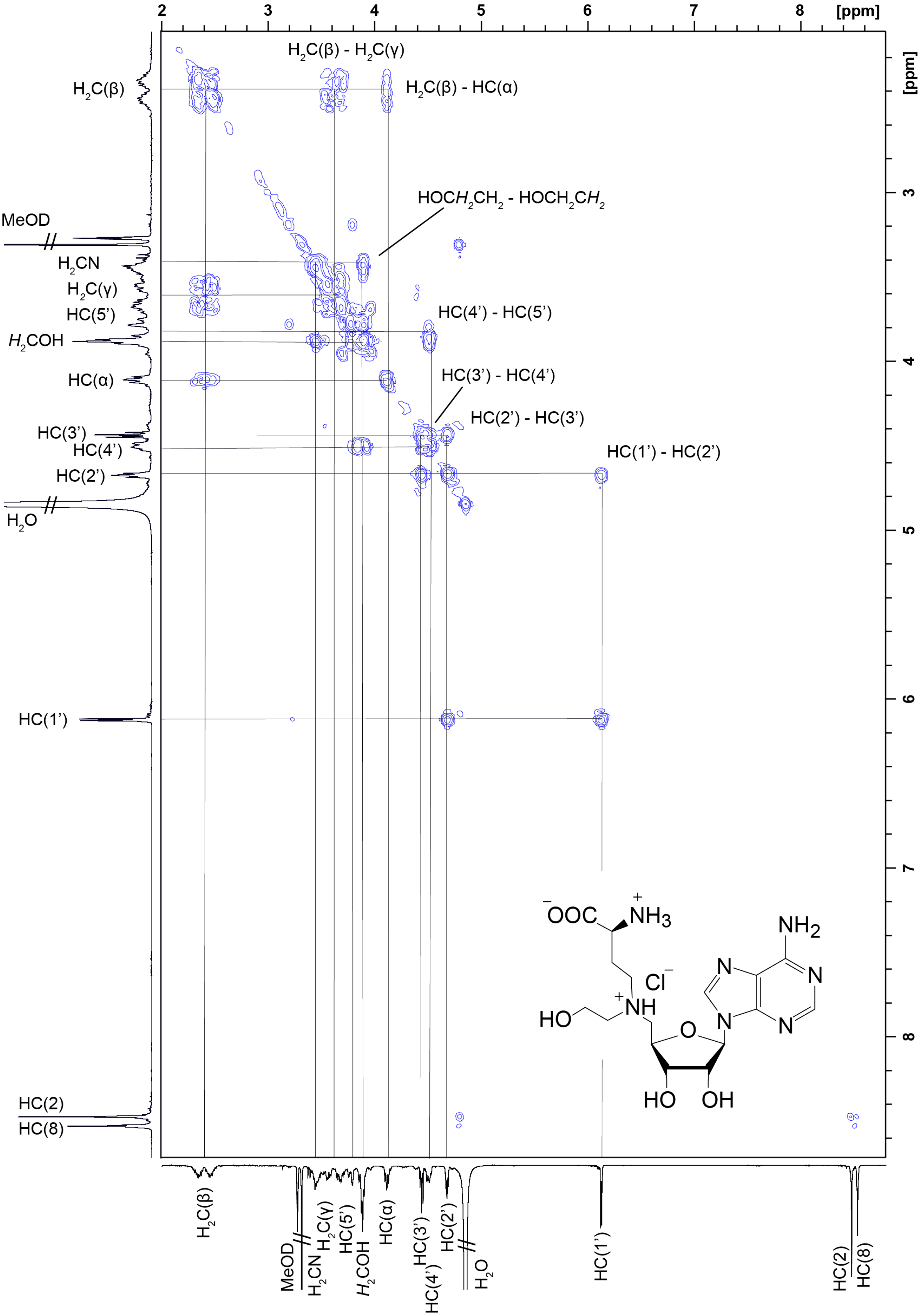


**Supplementary Figure 10 | NMR spectra of ligand analog M1.** $^1H/^1H$ COSY NMR spectrum (400 MHz, MeOD).

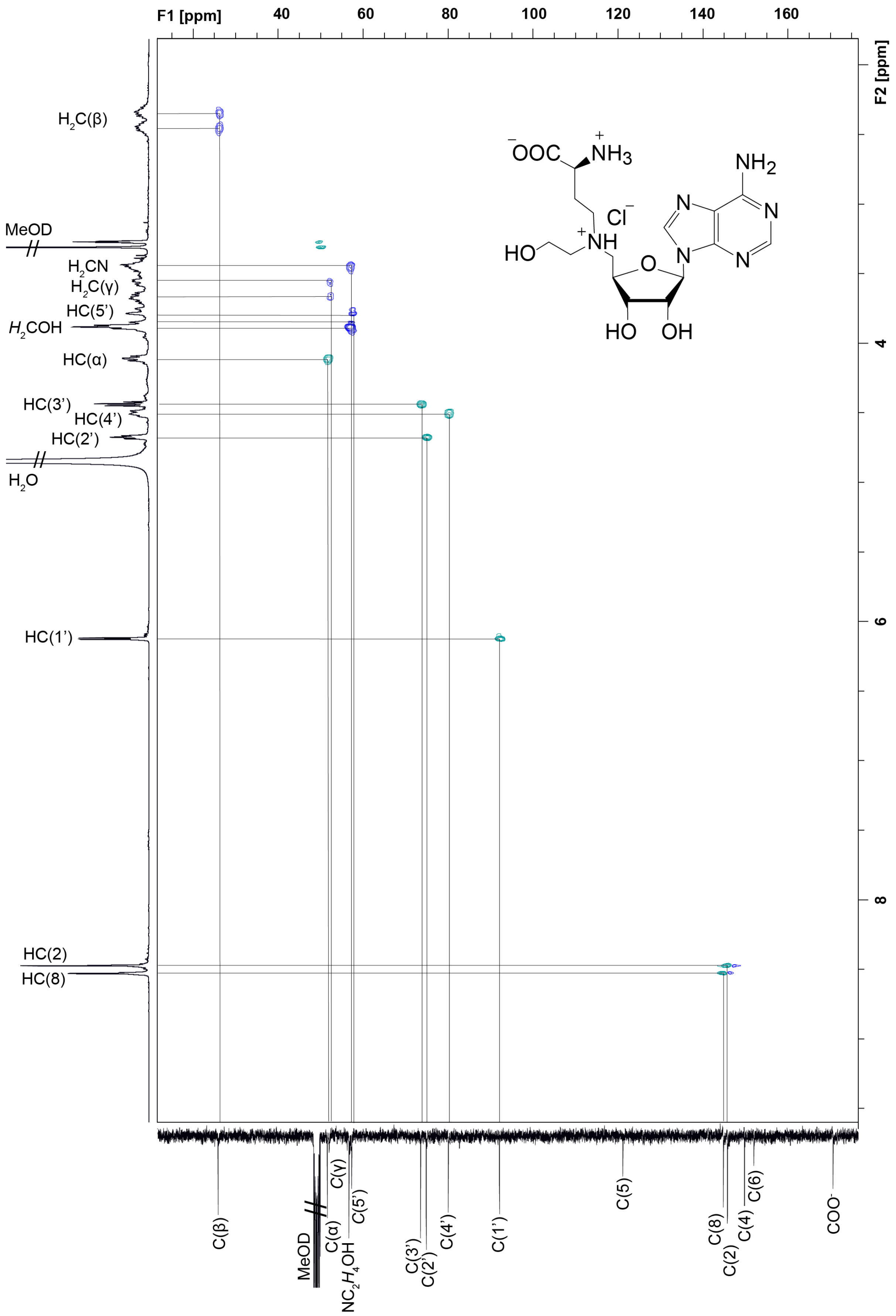


**Supplementary Figure 11 | NMR spectra of ligand analog M1.** $^{1}H/^{13}C$ HSQC NMR spectrum (400 MHz, MeOD).

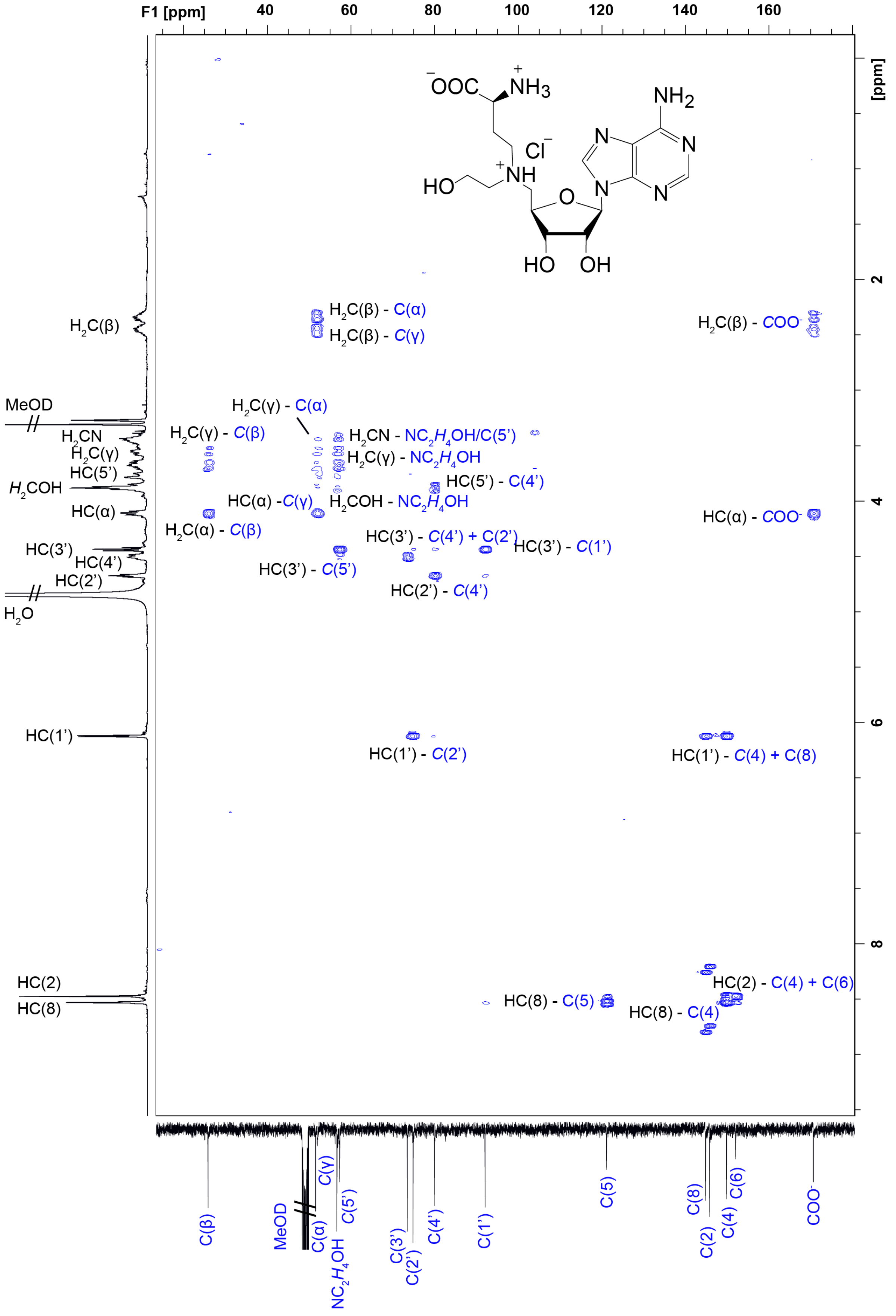


**Supplementary Figure 12 | NMR spectra of ligand analog M1.** $^{1}H/^{13}C$ HMBC NMR spectrum (400 MHz, MeOD).

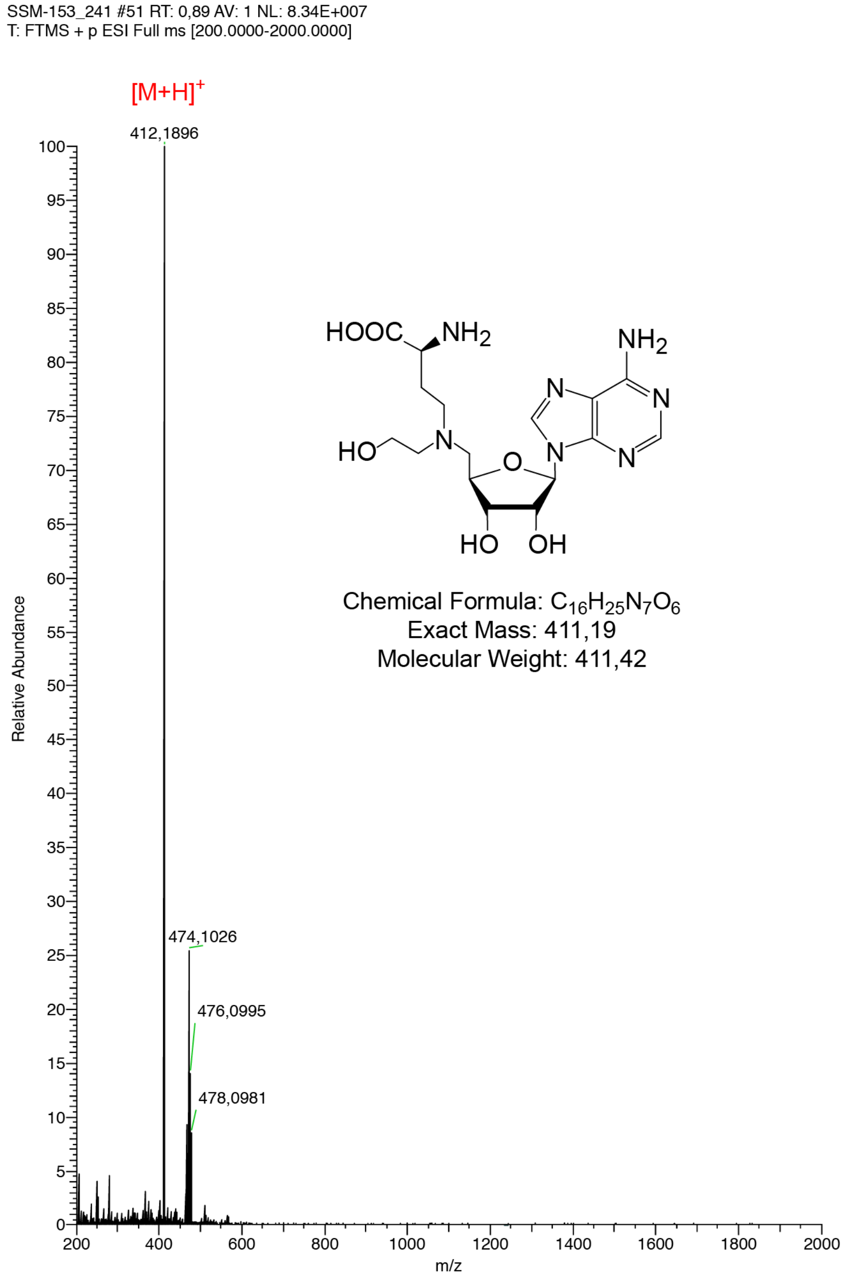


**Supplementary Figure 13 | Mass spectrometry of ligand analog M1.** Chemical structure and mass spectrum.

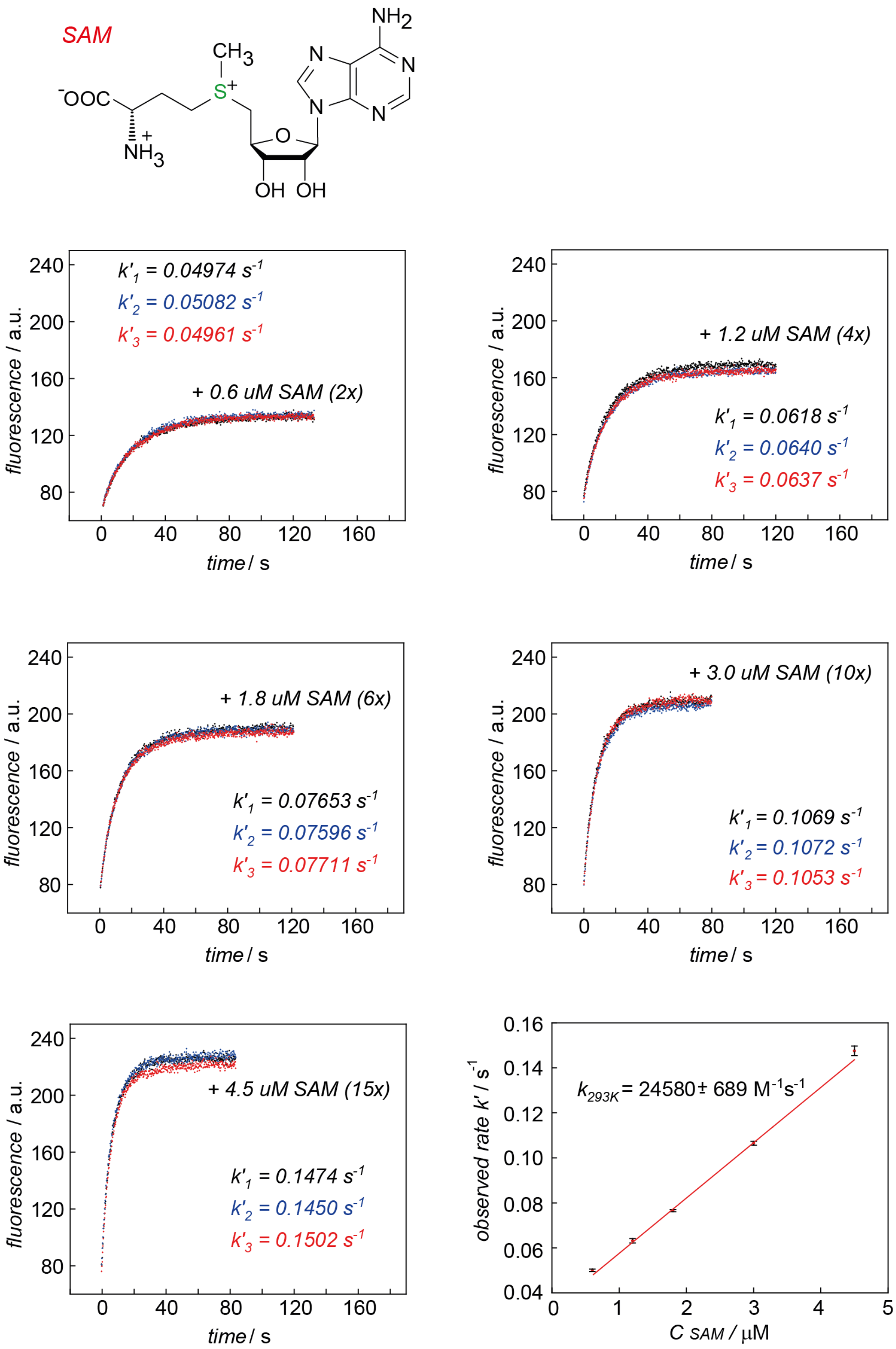


**Supplementary Figure 14 | Real time aminopurine (Ap) fluorescence time traces of the SAM-VI riboswitch complex formation (the wt U39Ap variant at different concentrations of SAM, ranging from 0.6 to 4.5 µM, pH 7.5).** Stopped-flow fluorescence spectroscopy was used to monitor the kinetics of SAM-VI riboswitch complex formation. The mean k' values determined from three independent experiments with the corresponding error bars are plotted against the concentration of SAM and subjected to a linear fit. The slope of the plot yields the rate constant $k_{293K}$. Conditions: $C_{RNA}$ = 0.3 µM, 50 mM KMOPS, 100 mM KCl, 2 mM $MgCl_2$, 293 K. Further details are available in Methods section.

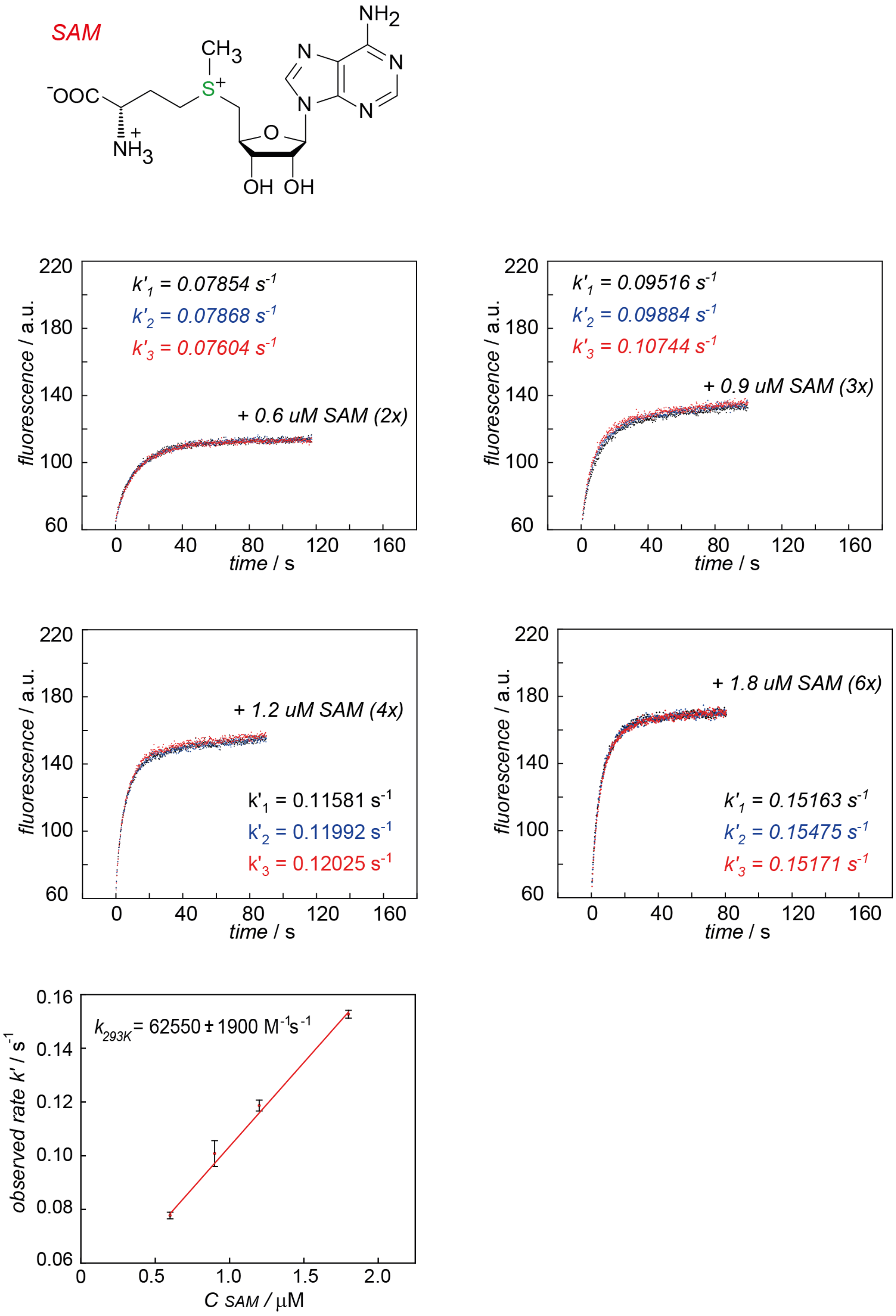


**Supplementary Figure 15 | Real time aminopurine (Ap) fluorescence time traces of the SAM-VI riboswitch complex formation (the U6C U39Ap variant at different concentrations of SAM, ranging from 0.6 to 1.8 µM, pH 7.5).** For conditions and further details, see Methods and the caption for Supplementary Figure 14.

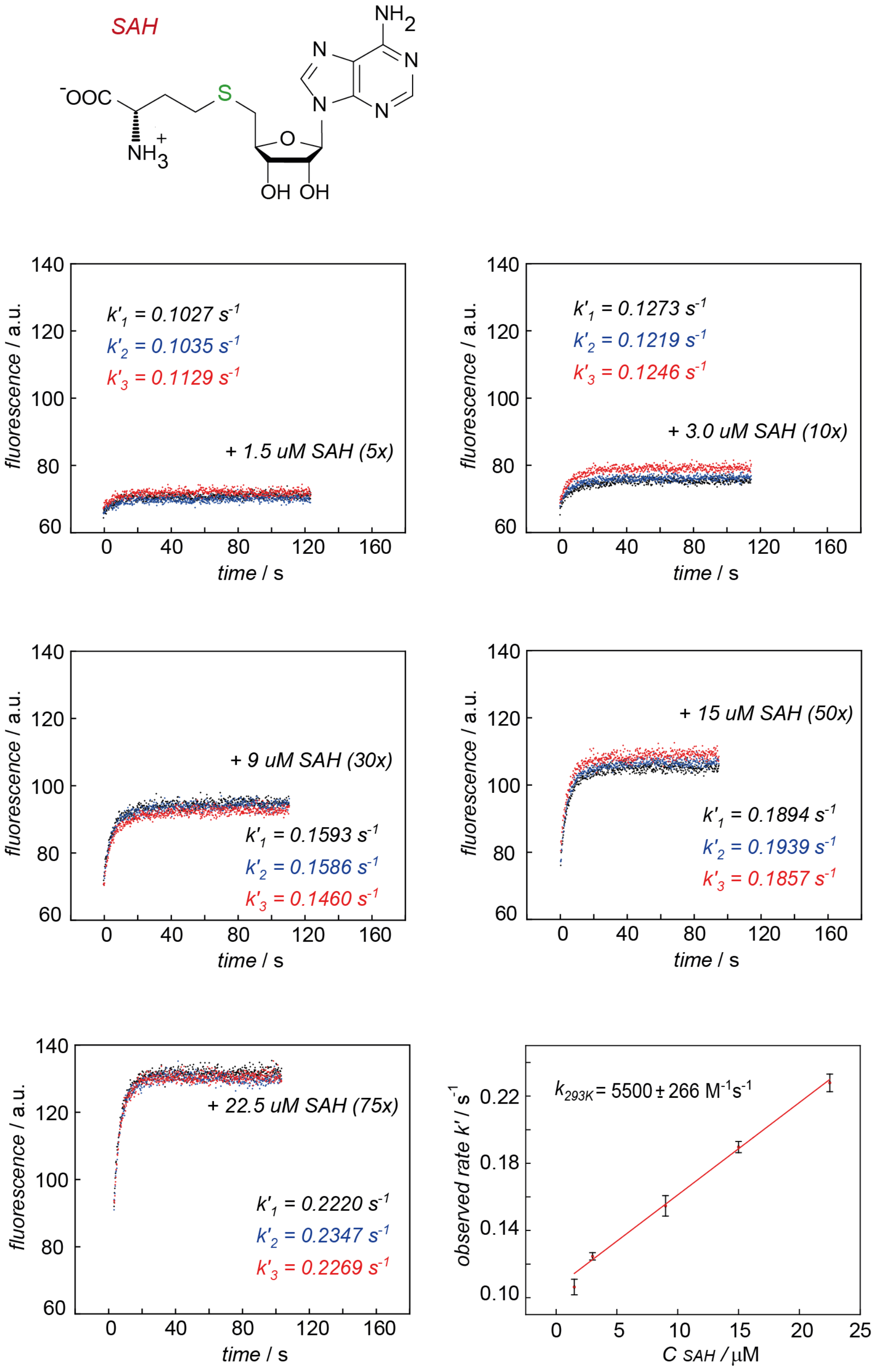


**Supplementary Figure 16 | Real time aminopurine (Ap) fluorescence time traces for the SAM-VI riboswitch complex formation (the wt U39Ap variant at different concentrations of SAH, ranging from 1.5 to 22.5 µM, pH 7.5).** For conditions and further details, see Methods and the caption for Supplementary Figure 14.

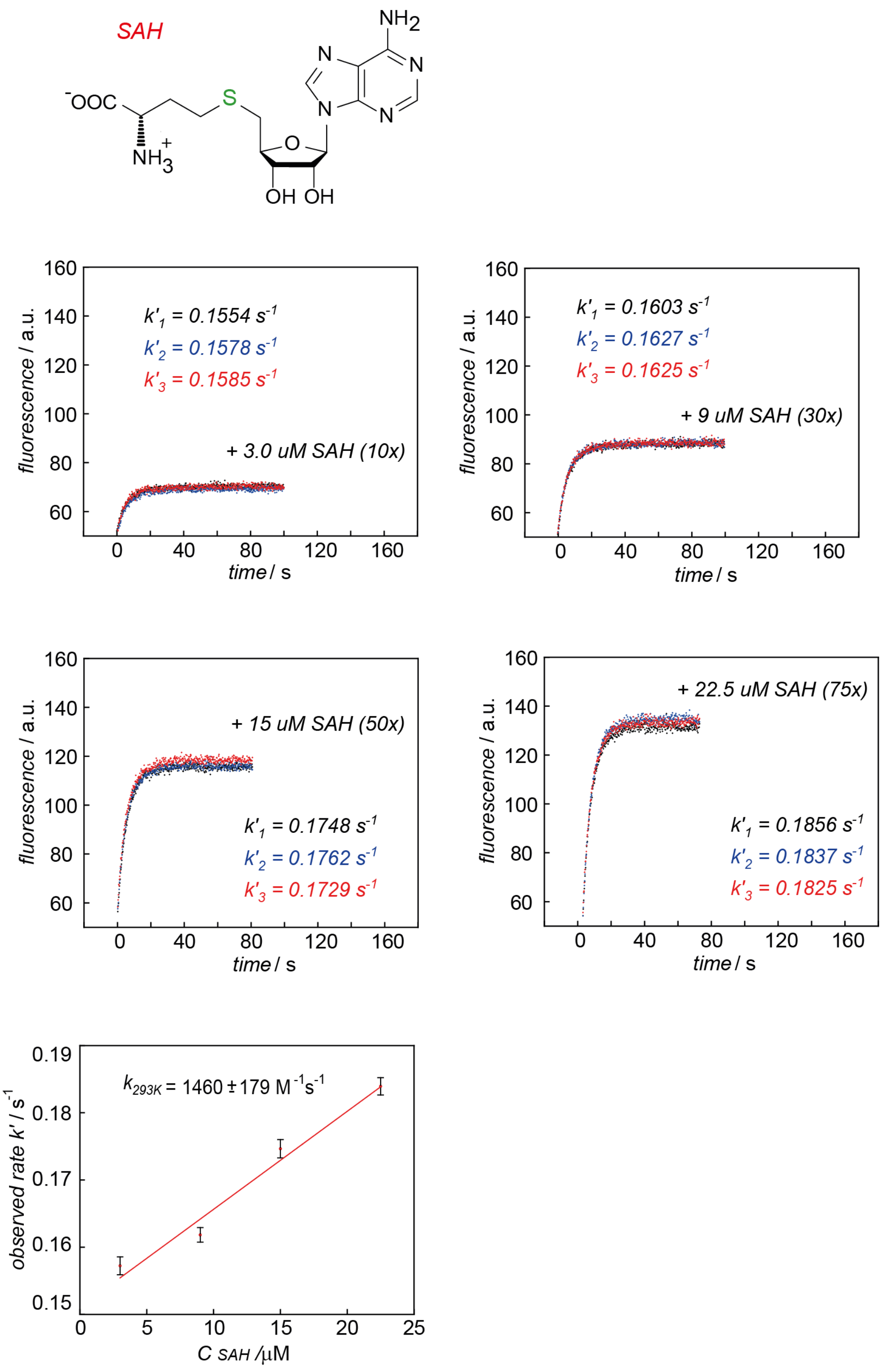


**Supplementary Figure 17 | Real time aminopurine (Ap) fluorescence time traces for the SAM-VI riboswitch complex formation (the U6C U39Ap variant at different concentrations of SAH, ranging from 3.0 to 22.5 µM, pH 7.5).** For conditions and further details, see Methods and the caption for Supplementary Figure 14.

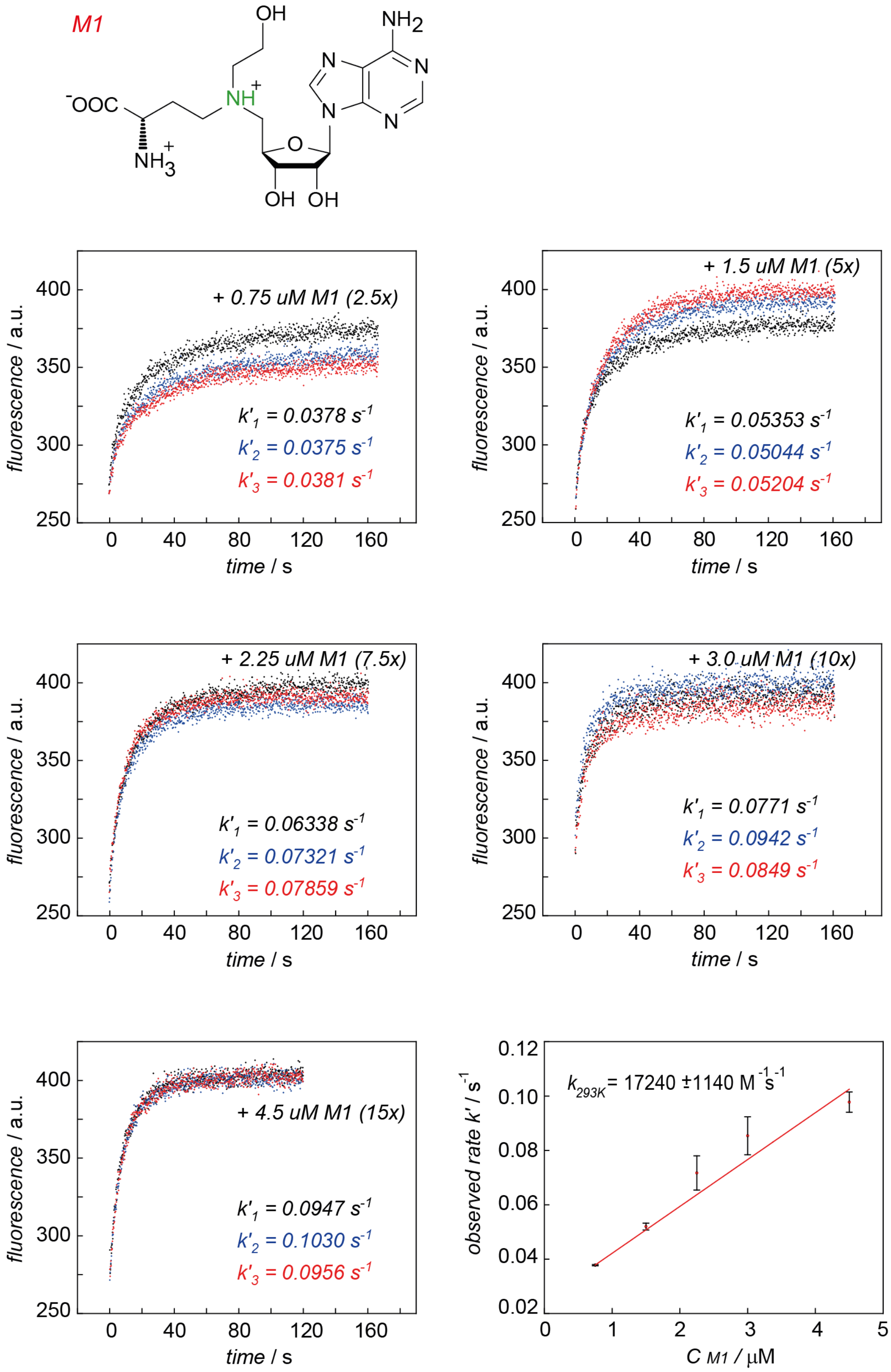


**Supplementary Figure 18 | Real time aminopurine (Ap) fluorescence time traces for the SAM-VI riboswitch complex formation (the wt U39Ap variant at different concentrations of the M1 ligand, ranging from 0.75 to 4.5 µM pH 7.5).** For conditions and further details, see Methods and the caption for Supplementary Figure 14.

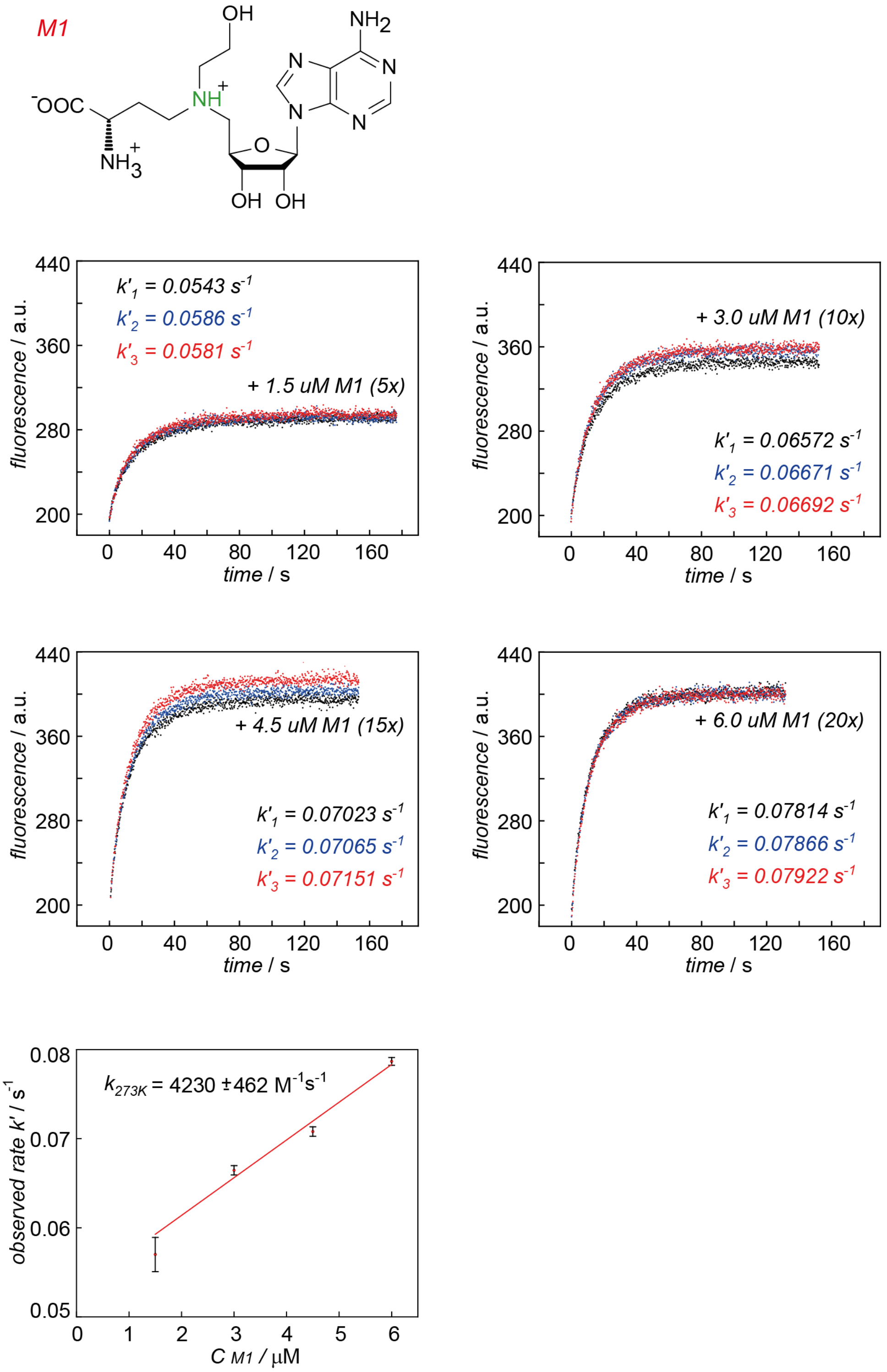


**Supplementary Figure 19 | Real time aminopurine (Ap) fluorescence time traces for the SAM-VI riboswitch complex formation (the U6C U39Ap variant at different concentrations of the M1 ligand, ranging from 1.5 to 6.0 μM, pH 7.5).** For conditions and further details see Methods and the caption for Supplementary Figure 14.

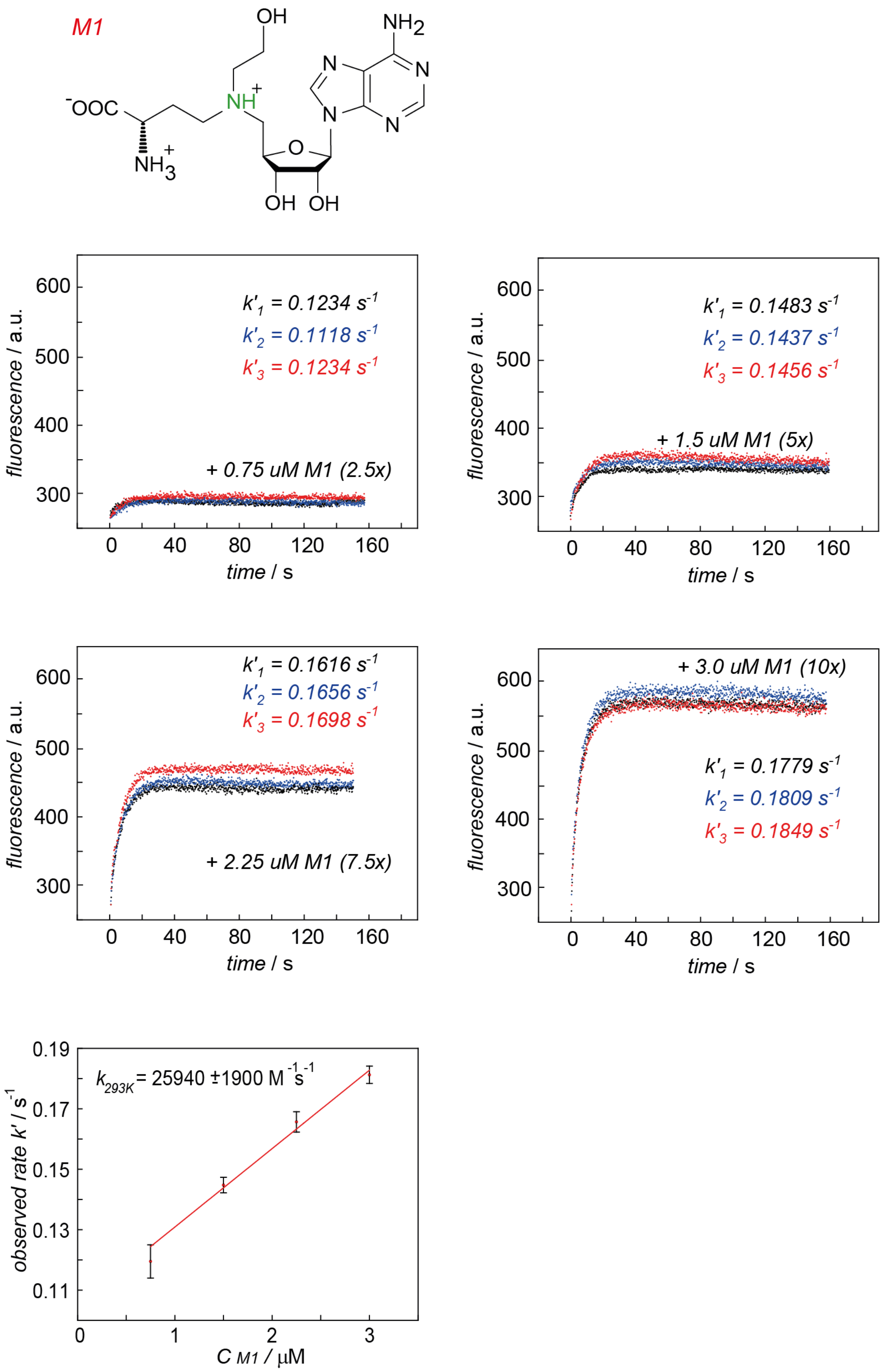


**Supplementary Figure 20 | Real time aminopurine (Ap) fluorescence time traces for the SAM-VI riboswitch complex formation (the wt U39Ap variant at different concentrations of the M1 ligand, ranging from 0.75 to 3.0 µM, pH 6.0).** For conditions and further details, see Methods and the caption for Supplementary Figure 14.

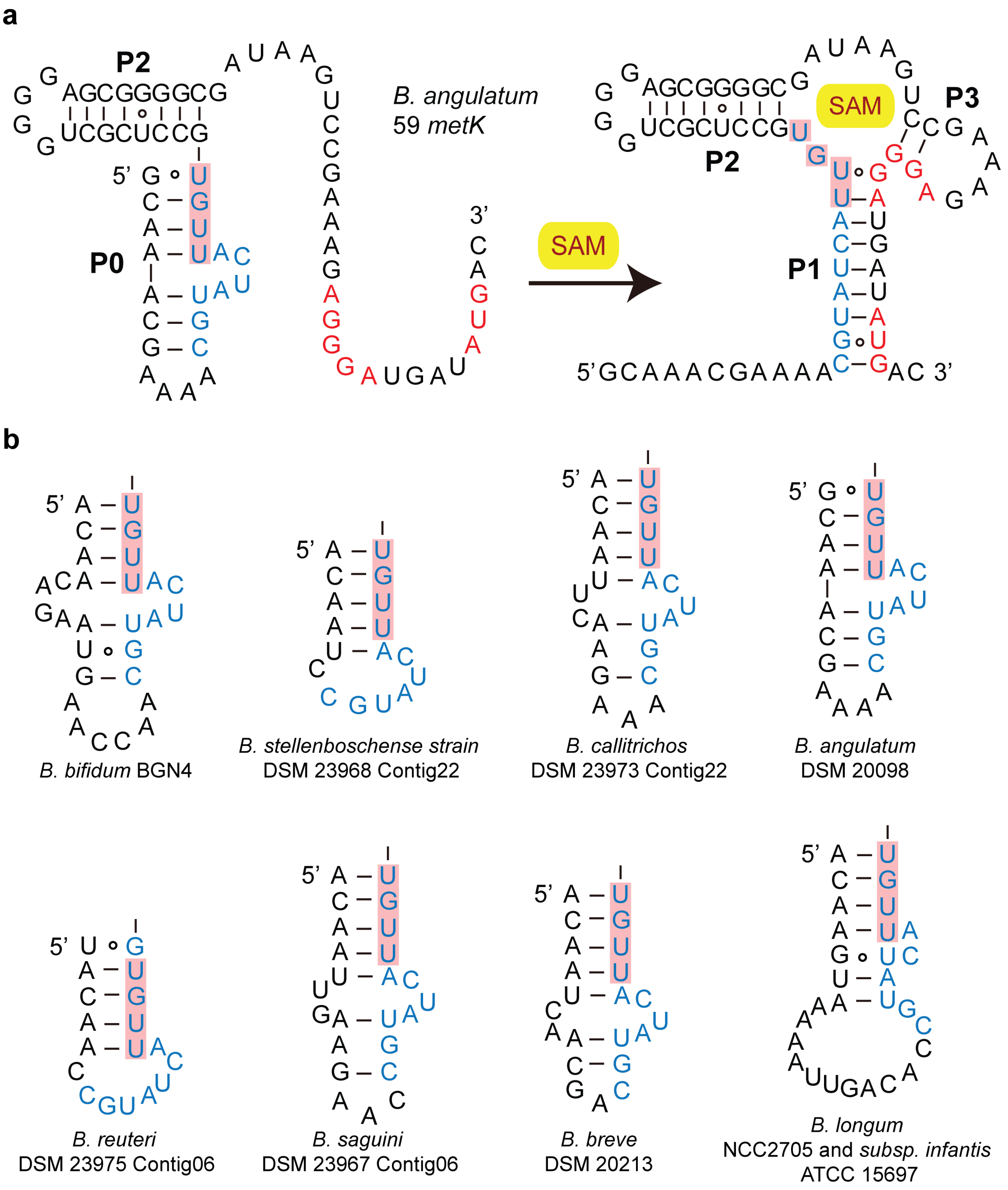


**Supplementary Figure 21 | Model for sequential folding of the nascent SAM-VI riboswitch RNA.** Stem P0 forms early in the path and assists in blocking the P1-P2 junctional region (shaded in red), hence supporting ribosomal access and recognition of the Shine-Dalgarno (SD) sequence. In the presence of high SAM concentrations the junctional fold is captured and stabilized by ligand binding, blocking the SD sequence and in turn, preventing ribosomal recognition and shutting off translation.

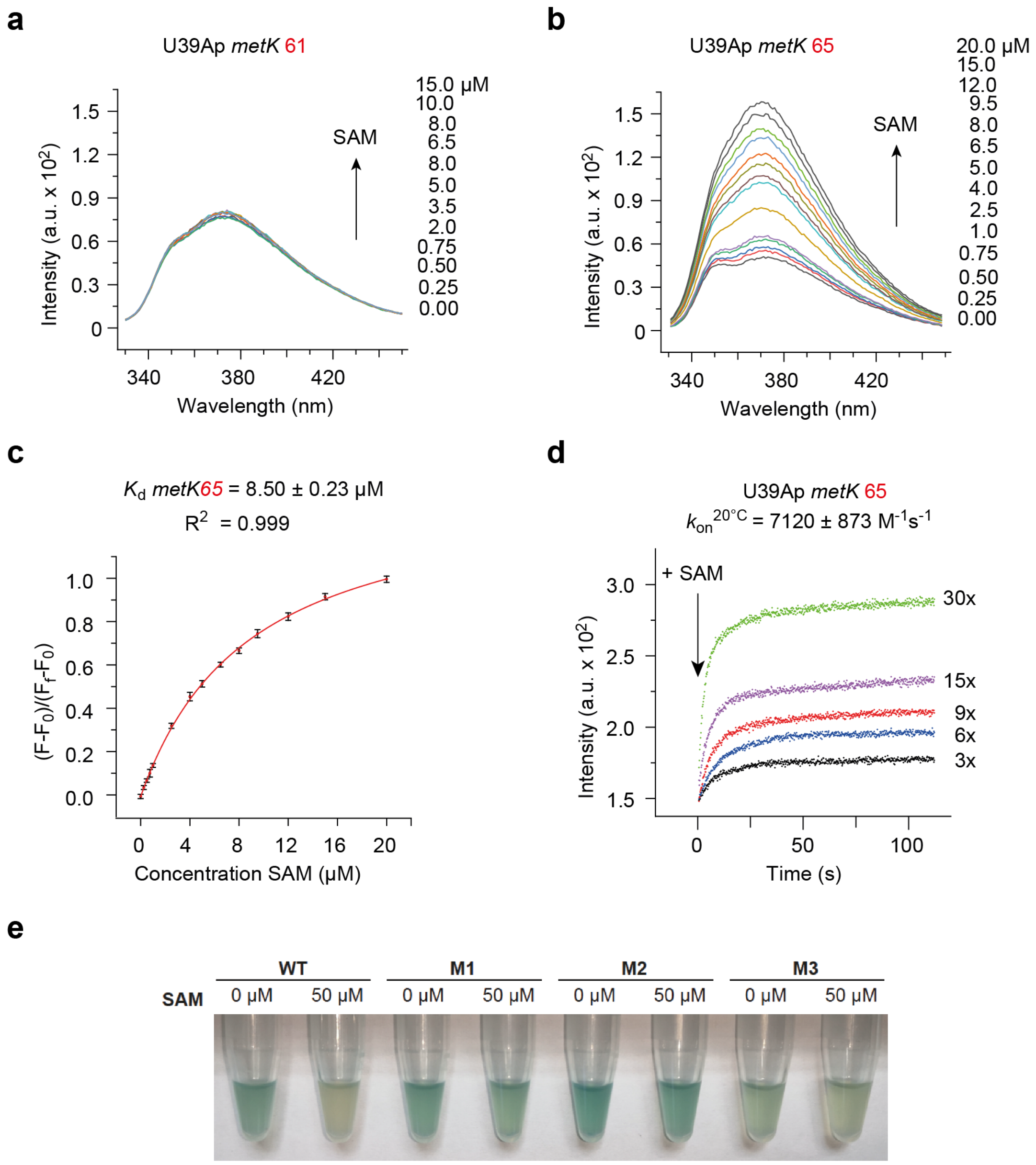


**Supplementary Figure 22 | Model for sequential folding and translational gene control of the SAM-VI riboswitch. a**, Fluorescence changes upon titration of U39Ap labeled SAM-VI riboswitch *metK* 61 with ligand SAM; fluorescence emission spectra ($\lambda_{ex}$ = 308 nm) from 330 to 450 nm of the U39Ap variant for each ligand concentration. b, Same as a but for U39Ap labeled SAM-VI riboswitch *metK* 65. **c**, Normalized fluorescence intensity of *metK* 65 RNA plotted as a function of SAM ligand concentration. The graph shows the best fit to a single-site binding model (see Methods). Changes in fluorescence ($F$-$F_0$) were normalized to the maximum fluorescence measured in saturating concentrations of the SAM ligand. Conditions: c(RNA) = 0.5 μM; 50 mM KMOPS, pH 7.5, 100 mM KCl, 2 mM $MgCl_2$, 293 K. d, Fluorescence spectroscopy was used to monitor the kinetics of of *metK* 65 RNA–SAM complex formation. Exemplary fluorescence traces are depicted; conditions: 0.3 μM RNA, 100 mM KCl, 50 mM MOPS, pH 7.5, 293 K. Ligand concentrations as indicated. **d**, Qualitative β-galactosidase assays (for details see Figure 5 and Methods).
